# Genetic variability, cryptic species and phylogenetic relationship of six cyathostomin species based on mitochondrial and nuclear sequences

**DOI:** 10.1038/s41598-021-87500-8

**Published:** 2021-04-15

**Authors:** Mariana Louro, Tetiana A. Kuzmina, Christina M. Bredtmann, Irina Diekmann, Luís M. Madeira de Carvalho, Georg von Samson-Himmelstjerna, Jürgen Krücken

**Affiliations:** 1 Institute for Parasitology and Tropical Veterinary Medicine, Department of Veterinary Medicine, Freie Universität Berlin, Berlin, Germany; 2 CIISA – Centre for Interdisciplinary Research in Animal Health, Faculty of Veterinary Medicine, University of Lisbon, Lisbon, Portugal; 3 I. I. Schmalhausen Institute of Zoology, National Academy of Sciences of Ukraine, Kyiv, Ukraine

**Keywords:** Parasite genetics, Parasite evolution

## Abstract

Cyathostomins are important intestinal nematode parasites of equines and include 50 accepted species. Their taxonomy has been frequently revised and the presence of cryptic species suggested. Furthermore, usually molecular- and morphology-based phylogenetic analyses give divergent results. In this study, the nucleotide sequences of the nuclear second internal transcribed spacer (ITS-2) and the mitochondrial partial cytochrome c oxidase subunit I (COI) were determined for adults of six cyathostomin species (*Coronocyclus coronatus*, *Coronocyclus labiatus*, *Cylicocyclus nassatus*, *Cylicostephanus calicatus, Cylicostephanus longibursatus*, *Cylicostephanus minutus*) collected from different equine species within two geographic regions. Maximum likelihood trees were calculated for ITS-2, COI, and concatenated data. No obvious differentiation was observed between geographic regions or equine host species. As previously reported, *Coronocyclus coronatus* and *Cylicostephanus calicatus* revealed a close relationship. Cryptic species were detected in *Cylicostephanus minutus* and *Cylicostephanus calicatus*. *Cylicocyclus nassatus* and *Coronocyclus labiatus* showed diverse mitochondrial and nuclear haplotypes occurring in different combinations, while *Cylicostephanus longibursatus* was comparatively homogenous. In conclusion, a combined analysis of nuclear and mitochondrial haplotypes improved resolution of the phylogeny and should be applied to the remaining cyathostomin species and across additional equine host species and geographic regions.

## Introduction

Worldwide, equines are exposed to intestinal helminth infections that can compromise their health and welfare. The biodiversity of parasitic nematode species in horses is ample, but the great majority belongs to the family Strongylidae. The species from this family are divided in two subfamilies, Strongylinae (strongylins) and Cyathostominae (cyathostomins), which, among other criteria, differ morphologically by the form of the buccal capsule and to some extent, by their respective size^1^. Although Strongylidae comprise the most pathogenic nematodes of horses, information on these parasites to date focuses on characteristics of morphology, life cycle, prevalence, and disease control and prevention. Data are particularly limited concerning the genetic and molecular characteristics of cyathostomins^2,3^.

Cyathostomins are currently considered the most important horse nematodes^4^, especially due to the reduced prevalence of *Strongylus* spp. and the spread of cyathostomin populations with phenotypic anthelmintic resistance^5^. They occur ubiquitously and inhabit the ventral and dorsal colon and the caecum of infected equines with high prevalence and abundance. Most equines can harbour thousands of cyathostomins without developing clinical disease, but, in some cases, they can lead to a clinical syndrome called “larval cyathostominosis” with a reportedly 50% fatality rate^6^. The subfamily Cyathostominae contains 50 accepted species^1^, but only 13 species probably account for 98–99% of the total cyathostomin burden worldwide^7–10^: *Cylicocyclus (Cyc.) nassatus, Cyc. ashworthi, Cyc. leptostomum, Cyc. insigne, Cyathostomum (Cya.) catinatum, Cya. pateratum, Cylicostephanus (Cys.) longibursatus, Cyc. goldi, Cys. minutus, Cys. calicatus, Coronocyclus (Cor.) coronatus, Cor. labiatus,* and *Cor. labratus.* However, due to several problems in identification of cyathostomins to the species level summarized by Bredtmann et al.^11^, little is known about the biology and ecology of individual species or how species interact with one another in the host or in the external environment. The accurate identification of cyathostomins is crucial in order to study their biology, epidemiology, and pathogenicity as well as their current drug susceptibility status. Most species-specific data are derived from adult specimens collected at post-mortem examinations or collected after anthelmintic treatments, and are often complicated by the difficulties in accurate microscopic identification of specimens, which requires an expert morphologist with long-term training and continuous experience. Additionally, species-specific morphologic identification of cyathostomins is not possible for other life cycle stages^12–14^. To further complicate the situation, the validity of some cyathostomin species is questionable, and others might represent cryptic species complexes^15,16^. To overcome the limitation of morphological identification, diverse techniques have been applied to study cyathostomins, such as serological^17–19^ and common molecular methods^20–24^ as well as, more recently, proteomic approaches^25^. This study applies molecular methods to assess the utility of two different genes—the nuclear second internal transcribed spacer (ITS-2) and the mitochondrial partial cytochrome c oxidase subunit I (COI)—as barcode regions and the phylogenetic relationships of six common species of cyathostomins (*Cor. coronatus*, *Cor. labiatus*, *Cyc. nassatus*, *Cys. calicatus, Cys. longibursatus*, *Cys. minutus*). While for two pairs of species, previous publications have presented partial data sets^24^, the present study includes two additional species, adds data derived from additional specimens for the previously studied species, and, most importantly, provides a complete analysis of intra- and interspecies variability over these six recognized species.

## Results

### Samples, PCR amplification, and molecular cloning

In total, 349 cyathostomin specimens (279 from Ukraine and 70 from Germany) were included in this study and represented six species: *Coronocyclus coronatus* (n = 60), *Cor. labiatus* (n = 60), *Cyc. nassatus* (n = 60), *Cys. calicatus* (n = 63), *Cys longibursatus* (n = 54), and *Cys. minutus* (n = 52). Regarding the hosts, 70 specimens were collected from eight German domestic horses (*Equus ferus caballus*), while from equines kept at Askania Nova Biosphere reserve, Ukraine, 54 worms were collected from a donkey (*Equus africanus asinus*), 57 from a domestic horse (*Equus ferus caballus*), 60 from a Kulan (*Equus hemionus kulan*), 60 from a Przewalski's horse (*Equus ferus prezewalskii*), and 48 from a plains zebra (*Equus quagga burchelli*) (Table S1). DNA was successfully extracted from all cyathostomin specimens.

ITS-2 amplification was successful for all specimens. For all *Cor. coronatus*, *Cor. labiatus,* and *Cys. longibursatus* specimens, 278 bp, 368 bp, and 370 bp fragments were amplified, respectively. For some *Cor. coronatus* an additional PCR product of an additional 369–370 bp was obtained as described previously^24^. This fragment was not included in the phylogenetic analysis since it was only present in a subset of the specimens. Amplification of DNA extracted from 46 *Cys. calicatus* specimens resulted in 369–370 bp fragments while for 17 specimens 281 bp fragments were obtained. For *Cyc. nassatus*, amplification of DNA from 59 specimens generated 370 bp fragments while for one specimen a 365 bp fragment was encountered. For *Cys. minutus,* the ITS-2 PCR for 51 specimens gave 265 bp fragments and, for one specimen, a 266 fragment.

COI amplification was successful for 337 specimens. All amplified samples consisted of 653 bp fragments, corresponding to 217 amino acids, and the absence of indels or in frame stop codons suggests that the sequence quality was high. The 12 unsuccessfully amplified specimens belonged to *Cor. coronatus* (n = 1), *Cyc. nassatus* (n = 1), and *Cys. calicatus* (n = 10) and were excluded from the COI analysis, including the tree calculated from concatenated data.

### Substitution saturation analyses

The COI alignment was split into one alignment containing codon positions 1 and 2 and another containing the codon position 3. The two alignments and the ITS-2 alignment were then analyzed independently for substitution saturation. Initially, the frequencies of transitions and transversions was plotted versus the Jukes-Cantor genetic distance for all pairs of sequences in the alignment (Fig. S1). A quadratic regression curve was used to show the trend. For ITS-2 (Figure S1A, D) and first and second codon positions of COI (Figure S1B, E), the frequencies of transitions were well above frequencies of transversions for all observed genetic distances indicating that there was no substitution saturation. In contrast, some substitution saturation was observed at high genetic distances when numbers of transitions did not increase further while transversion frequency continued to increase (Figure S1C). In order to determine if there was enough phylogenetic signal in the alignment, substitution saturation tests were conducted. The results of these tests showed that for ITS-2 and the first and second codon position of the COI gene the index for substitution saturation Iss was significant lower than the critical index for substitution saturation (Iss.c) independently of the tree topology. For the third codon position of COI, this was also the case for a symmetrical tree but not for the most extreme asymmetrical tree topology (Table S2).

### Presentation of phylogentic trees

**Figure 1:**
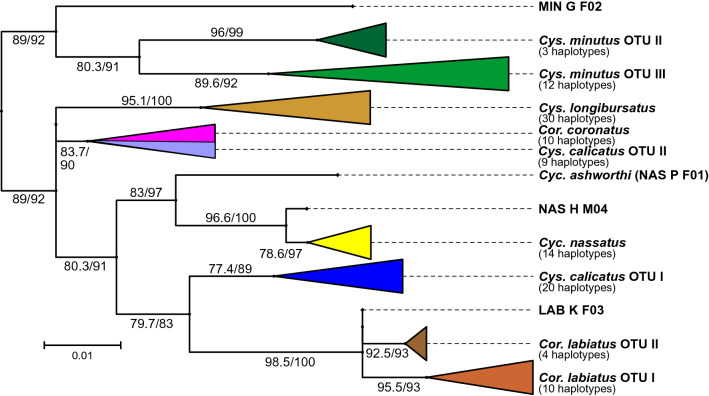
Maximum likelihood phylogenetic tree calculated using aligned ITS-2 sequences after condensing. The tree was manually rooted placing *Cylicostephanus minutus* at the base of the tree due to previous analysis on a larger number of species^55^. The scale bar represents 0.01 substitutions per site and node support was obtained by ultrafast bootstrapping (1000 replicates) after and the Shimodaira-Hasegawa likelihood ratio test before the slash. Individual specimens that were not assigned to one of the major clades are designated according to the following code: MIN, NAS, LAB for the species *Cylicostephanus minutus, Cylicocyclus nassatus, Coronocyclus labiatus*, respectively; G, H, P, K, D, Z for the hosts German horse, Ukrainian horse, Przewalski’s horse, kulan, donkey and zebra, respectively; F/M for female or male; a number indicating the individual specimen. OTU, operational taxonomic unit.

The substitution models with all model parameters are provided in Table S3. All phylogenetic trees were compressed to their major clusters to allow better visualization. In most of the compressed clusters, there was evidence of the presence of several haplotypes (numbers given in Fig. 1); however, this project focused on the major differences found within each species and the comparison between different cyathostomin species. For more details regarding the relationship of individual sequences within the clusters, complete trees are provided in Nexus format in Data S1 to S3.

### Internal transcribed spacer 2 phylogenetic tree

Most of the specimens grouped by species (Fig. 1). Within a highly supported *Cys. minutus* cluster, three distinct subclusters were formed, which were also supported by high bootstrap values as described previously using only *Cys. longibursatus* as outgroup^24^. The first, with only one sequence, the second, with 13 sequences, and the third with 38 sequences, were subclusters considered cryptic species and designated operational taxonomical units (OTU) I, II and III, respectively, by Bredtmann et al.^24^. *Cylicocylus nassatus* specimens formed three distinct clusters. The most basal of these clusters corresponded to the only specimen with the shorter sequence and was found to be identical to GenBank accession no. Y08586 assigned to *Cyc. ashworthi.* The other clusters contained one specimen and the remaining 58 specimens, respectively. A polytomy appeared on *Cor. labiatus* with three cluster. The first cluster contained only one sequence, the second comprised a larger cluster with 45 sequences, which, according to the following COI analysis were designated as OTU I, and the third cluster was smaller with 14 sequences designated as OTU II. Comparatively, *Cys. longibursatus* was more homogenous and presented a single cluster. *Coronocyclus coronatus* sequences grouped as a single cluster with the 17 smaller sequences of *Cys. calicatus* as described previously^24^. These 17 *Cys. calicatus* sequences were considered OTU II, while the remaining 46 larger sequences were considered OTU I, since they appeared together in a distinct branch with three species with *Cyc. ashworthi*, and *Cyc. nassatus* located between the two *Cys. calicatus* clusters.

### Cytochrome c oxidase I phylogenetic tree

**Figure 2:**
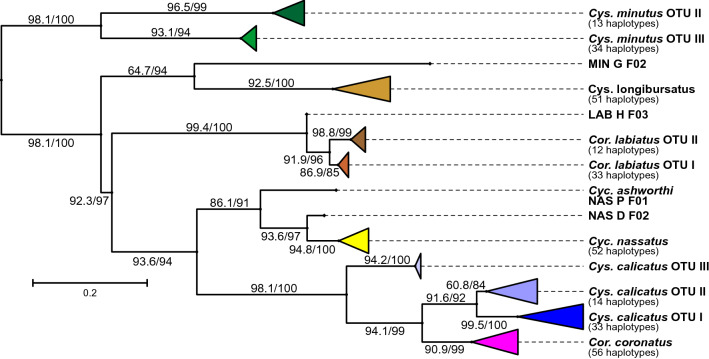
Maximum likelihood phylogenetic condensed tree calculated using aligned cytochrome oxidase 1. The scale bar represents 0.2 substitutions per site and node support was obtained by ultrafast bootstrapping (1000 replicates) after and the Shimodaira-Hasegawa likelihood ratio test before the slash. Individual specimens that were not assigned to one of the major clades are designated according to the following code: MIN, NAS, LAB for the species *Cylicostephanus minutus*, *Cylicocyclus nassatus*, *Coronocyclus labiatus*, respectively; G, H, P, K, D, Z for the hosts German horse, Ukrainian horse, Przewalski’s horse, kulan, donkey and zebra, respectively; F/M for female or male; a number indicating the individual specimen. OTU, operational taxonomic unit.

The obtained phylogenetic tree did not show an extreme asymmetric topology suggesting that use of the data from the third codon position was acceptable. Even though similar to the ITS-2 phylogenetic tree, there were notable differences and more heterogeneity (Fig. 2). For *Cys. minutus* the same clades appeared containing the same specimens. However, there were two specimens that were assigned to OTU II in the ITS-2 and OTU III in the COI analysis (MIN_Z_F03 and MIN_Z_F02). A similar effect occurred for *Cyc. nassatus*, where two specimens were assigned to different clusters in ITS-2 and COI analyses, but the one identified later as *Cyc. ashworthi* remained apart. *Coronocyclus labiatus* was organized in three distinct clusters, but all were composed of different specimens compared to the ITS-2 tree. The OTU I cluster was smaller than the OTU II cluster, which was reciprocal in the ITS-2 tree. *Cylicostephanus calicatus* sequences formed three distinct clusters: one was composed of all the 17 specimens with shorter ITS-2 sequences (OTU II), a branch with two sequences (OTU III) and the remaining sequences as OTU I. *Coronocyclus coronatus* formed a single cluster but appeared in the middle of the different *Cys. calicatus* clusters.

### Concatenated ITS-2 and COI phylogenetic tree

**Figure 3:**
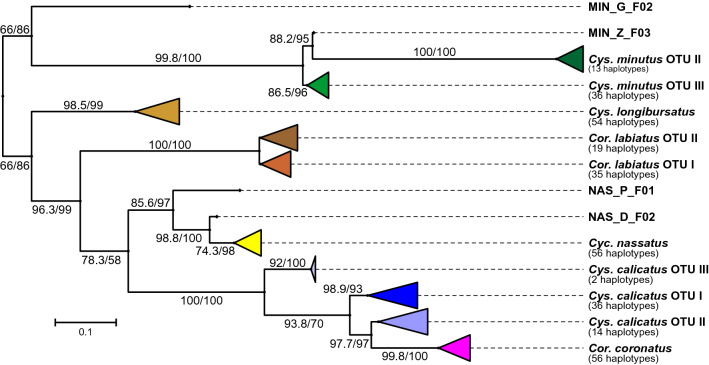
Maximum likelihood phylogenetic condensed tree calculated using aligned concatenated internal transcribed spacer 2 and cytochrome oxidase I sequences. The scale bar represents 0.1 substitutions per site and node support was obtained by ultrafast bootstrapping (1000 replicates) after and the Shimodaira-Hasegawa likelihood ratio test before the slash. Individual specimens that were not assigned to one of the major clades are designated according to the following code: MIN and NAS for the species *Cylicostephanus minutus* and *Cylicocyclus nassatus*, respectively; G, H, P, K, D, Z for the hosts German horse, Ukrainian horse, Przewalski’s horse, kulan, donkey and zebra, respectively; F/M for female or male; a number indicating the individual specimen. OTU, operational taxonomic unit.

Combining both ITS-2 and COI sequences within individual specimens helped to clarify several findings (Fig. 3). *Cylicostephanus minutus* presented three clusters with the exact same specimens from the COI tree but one specimen (MIN_Z_F03) appeared on an additional fourth branch, which was most closely related to OTU II supported by a high bootstrap value. This particular specimen is one of the two that was associated with different clusters when comparing the ITS-2 and COI trees, while the other remained in the same cluster found in the COI tree. For *Cyc. nassatus*, the specimen separation remained the same as in the previous COI tree. Regarding *Cor. labiatus*, two distinct clusters were formed, instead of the previous three clusters on both the ITS-2 and COI trees, and the OTU I cluster was bigger than that of OTU II. Even though different haplotypes were present too, *Cys. longibursatus* remained the most homogenous species when compared to the other species. *Coronocyclus coronatus* and *Cys. calicatus* presented identical sequence clusters to that of the COI tree.

### Sequence identity analysis

**Figure 4:**
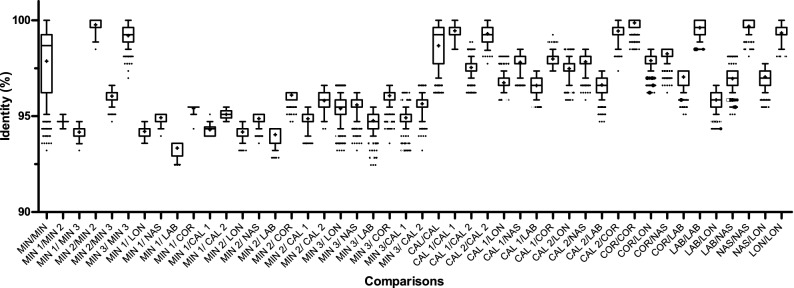
Comparison of sequence identity between different species and operational taxonomical units (OTU) on the internal transcribed spacer 2 sequences. Identities were calculated using dist.dna function from the ape package in R and plotted as boxplots (medians and 25%/75% percentiles) with whiskers showing the 95% percentiles and outliers shown by dots. Means are indicated by crosses. Abbreviations on the x-axis indicate the species *Coronocyclus coronatus* (COR), *Cylicocyclus nassatus* (NAS*), Cylicostephanus longibursatus* (LON), *Coronocyclus labiatus* (LAB), *Cylicostephanus calicatus* (CAL) and *Cylicostephanus minutus* (MIN). The OTUs of *Cys. calicatus* (OTU I and II) and *Cys. minutus* (OTU I, II, III) are represented as CAL 1, CAL 2 and MIN 1, MIN 2 and MIN 3, respectively.

**Figure 5:**
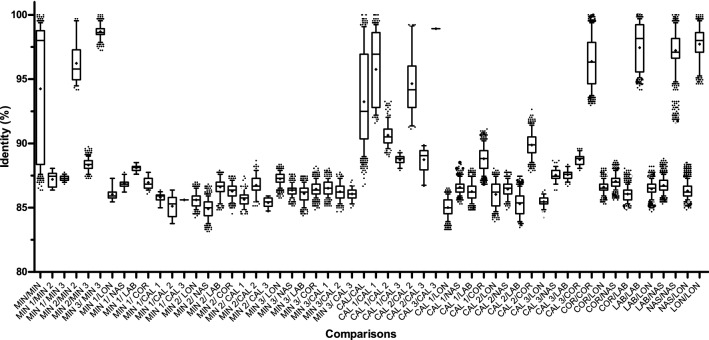
Comparison of sequence identity between different species and operational taxonomical units (OTU) on the cytochrome oxidase I sequences. Identities were calculated using dist.dna function and plotted as boxplots (medians and 25%/75% percentiles) with whiskers showing the 95% percentiles and outliers shown by dots. Means are shown as crosses. Abbreviations on the x-axis indicate the species *Coronocyclus coronatus* (COR), *Cylicocyclus nassatus* (NAS), *Cylicostephanus longibursatus* (LON), *Coronocyclus labiatus* (LAB), *Cylicostephanus calicatus* (CAL) and *Cylicostephanus minutus* (MIN). The OTUs of *Cys. calicatus* (OTU I, II and III) and *Cys. minutus* (OTU I, II, III) are represented as CAL 1, CAL 2, CAL 3 and MIN 1, MIN 2 and MIN 3, respectively.

The comparison of sequence identities between and within species and selected OTUs are shown in Fig. 4 for the ITS-2 sequences and in Fig. 5 for the COI sequences. A comparison within all sequences of each species was included. The OTUs from *Cys. calicatus* and *Cys. minutus* (from the combined COI and ITS-2 phylogenetic analysis) were considered distinct groups in each analysis and included in the comparison. The “*Cyc. nassatus*” sample identified as *Cyc. ashworthi* according to sequence data, was excluded from this analysis. Pairwise comparisons between all sequences belonging to *Cys. minutus*, revealed an intra-specific identity from 93.2 to 100% for the ITS-2 locus and from 86.9 to 100% for the COI locus, while within the three clusters it ranged from 97.0 to 100% and 94.2 to 100% for the ITS-2 and COI loci, respectively. In contrast, the identities between sequences of different *Cys. minutus* clusters ranged from 93.2 to 96.6% for the ITS-2 locus and from 86.4 to 89.7% for the COI locus. Pairwise comparisons between all sequences belonging to *Cys. calicatus* revealed an intra-specific identity for the ITS-2 and COI loci between 96.2–100% and 86.7–100%, respectively, while within the clusters it ranged from 94.2 to 100% for the ITS-2 locus and from 91.12 to 100% for the COI locus. The identities between sequences from the different *Cys. calicatus* clusters ranged from 96.2 to 98.9% and 86.7 to 93.3% at the ITS-2 and COI loci, respectively. The identities between *Cor. coronatus* sequences and the *Cys. calicatus* OTU II cluster ranged, for the ITS-2 locus, from 97.4 to 100% and, for the COI locus, from 87.8 to 92.7%. The intraspecific identities of sequences within the four remaining species (*Cor. coronatus*, *Cys. longibursatus*, *Cor. labiatus*, *Cyc. nassatus*) ranged at the ITS-2 locus from 98.1 to 100% and at the COI locus from 91.6 to 100%.

## Discussion

The COI gene is one of the most popular genetic markers and is commonly used as a barcode region for diverse species. For this study, the chosen primer set was specifically designed for amplification and sequencing of a 650 bp section of the COI gene in parasitic nematodes (excluding filaroid taxa)^26^. This primer pair was suitable to amplify most of the samples, and the sequences had the expected size (653 bp). The unsuccessful amplification of a few specimens could be due to differences in the sequence of the primer binding region, but low DNA quality or quantity must be considered as reasons. The mitochondrial genome of nematodes is known to have a high nucleotide substitution rate, which can lead to mutations on some more conserved regions of the COI gene such as the primer binding regions^27^. This hypothesis is supported by the observed variability in the present analysis, which is much higher for the mitochondrial gene compared to the nuclear ITS-2 sequence. Furthermore, the COI gene based phylogenetic analysis was able to differentiate all cyathostomin species tested in the present study, showing its usefulness as a barcode region. However, due to only maternal heritage^28^ resulting in the absence of recombination, mitochondrial marker sequences alone can give misleading results due to lineage sorting, since different mitochondrial genotypes can co-exist and do not recombine within the same species.

The ITS-2 gene is another barcode region widely used in many animal groups. Although amplification of the ITS-2 was more robust than that of the COI gene and was successful for all samples, it also was associated with analytical problems since PCR product sizes varied considerably which leads to difficulties in proper sequence alignment^29,30^. This variable sequence size occurred both within some of the species and between different species. The presence of polymorphism in individual *Cys. calicatus* worms complicates the use of this marker as a barcode region. ITS-2 sequences were not able to discriminate two species from different genera, *Cor. coronatus* and *Cys. calicatus*, confirming previous findings on the ruminant parasitic nematode genus *Cooperia*^31^ that the ITS-2 locus is not a fully reliable diagnostic marker on the species level. The addition of the variable mitochondrial marker COI enhanced the results of this analysis due to its superior barcoding properties^27^. Thus, with the combined analyses, *Cor. coronatus* and *Cys. calicatus* OTU II could be clearly separated, which was not the case with the ITS-2 sequences. However, even though the COI sequence-based analysis was able to clearly separate different species, it still put species from different genera closer together than species from within the same genus. Since assigning species within the subfamily Cyathostominae to a genus is based on overall similarities in morphological characters, it was expected that species within the same genus would be genetically more similar to each other than to species of other genera. While the phylograms produced from this molecular data are not entirely congruent with the current morphological classification^1^, the results are not surprising, since there are numerous examples of the incongruence between cyathostomin phylogenies based on molecular and morphological data sets^32–35^.

*Coronocyclus coronatus* and *Cys. calicatus* OTU II showed an obvious absence of distinct nuclear haplotypes on the ITS-2 phylogenetic tree and high identity values for the ITS-2 gene. By adding the COI gene to the analysis, differentiation of both species was clear. With the COI gene, several mitochondrial haplotypes for *Cor. coronatus* were observed as described recently^24^. However, the addition of more species to the analysis, including another from the genus *Coronocyclus* and two from the genus *Cylicostephanus*, did not alter the results previously found by Bredtmann et al.^24^ *Cor. coronatus* and *Cys. calicatus* remained closely related and the other *Cylicostephanus* species were in a position in the tree that was far away from these two and not in a common cluster with or even between *Cor. coronatus* and *Cys. calicatus*. Furthermore, *Cys. calicatus* sequences were organized in three distinct clusters, which had high intra-cluster identity values, favouring the hypothesis that they might represent cryptic species in a complex^24^. This hypothesis is also supported by the fact that all sequences from the nuclear ITS-2 *Cys. calicatus* OTU II came from the same set of individual specimens as those from the mitochondrial COI *Cys. calicatus* OTU II, indicating that nuclear and mitochondrial haplotype groups did not mix with other mitochondrial haplotypes, i.e. only particular combinations of nuclear and mitochondrial haplotype groups occurred while others were not observed. Hereupon, the COI marker has shown its usefulness as a barcode region by not only being able to clearly distinguish between morphologically different species but also by demonstrating evidence for the presence of cryptic species complexes.

Apart from two specimens (MIN_Z_F03 and MIN_Z_F02), the *Cys. minutus* samples did not mix between the distinct nuclear and mitochondrial clusters, i.e. fixed combinations of nuclear and mitochondrial haplotype groups occurred with the exception of the two before mentioned specimens. Furthermore, the identity values for both the ITS-2 and COI genes showed high values for comparisons within each *Cys. minutus* OTU and much lower values when comparing these OTUs with other groups of species. *Cys. minutus* OTU I was considered a valid and independent genospecies after several PCR and molecular cloning repetitions. These findings support the evidence for the presence of a cryptic species complex, as previously reported^15,25^. Regarding the two specimens that switched between nuclear and mitochondrial clusters it was unfortunately impossible to exclude contamination of PCRs due to depletion of the DNA samples. Thus the possibility of crossbreeding between both OTUs could neither be confirmed nor excluded.

An apparently morphologically misidentified *Cyc. nassatus* specimen was identified as *Cyc. ashworthi*, since its ITS-2 sequence matched one sample of this species with 99% of identity in GenBank (accession number Y08586^36^), and its COI sequence also matched unpublished *Cyc. ashworthi* samples from a part of the present project still in progress. As expected from the data published by Hung et al., the separation between these two species is evident in all phylogenetic trees^36^. *Cylicocyclus nassatus* and *Cyc. ashworthi* were historically considered as a single species and, even with updated morphological identification keys, are frequently misidentified^37^.

*Cylicocyclus nassatus* presented two distinct haplotype groups for both the nuclear and mitochondrial sequences. However, nuclear and mitochondrial haplotype groups apparently mixed freely with each other, i.e. all combinations of nuclear and mitochondrial haplotype groups were observed, confirming the absence of a cryptic species complex. Also, in contrast to a previous report, no obvious differentiation between geographic regions (Ukraine vs. Germany) or equine host species was found for *Cyc. nassatus*^16^. This absence of differentiation extends to all the other cyathostomin species in the present study, since larger clades contained specimens from both regions and more than one host species. The small number of clades with only one or two specimens does not allow any conclusions regarding geographic distribution or host specificity.

Like *Cyc. nassatus*, *Cor. labiatus* showed free mixing of distinct nuclear and mitochondrial haplotypes. Together with the higher sequence identities and small range of identities within this species compared to within *Cys. minutus* or within *Cys. calicatus*, these data revealed no evidence for the presence of a cryptic species complex.

The species with the lowest intra-species variability was *Cys. longibursatus*. The absence of distinct mitochondrial and nuclear haplotype groups suggests that it is a well-defined species based on both morphologic and genomic characters.

The observed patterns in the phylogenetic trees can be subject to several processes that might lead to wrong interpretation. Incomplete lineage sorting and hybridization between closely related species could lead to complex patterns in phylogenetic trees. However, the study design used with multiple specimens collected from several hosts should be robust regarding the misinterpretation of hybrids that should also have very distinct positions in multi-locus analyses. Disagreeing results between markers for specimens that were discussed above to be explainable by rare mixing of nuclear and mitochondrial haplotypes or PCR contaminations might in fact also represent hybridization between different species (as e.g. observed for MIN_Z_F03 and MIN_Z_F02). In fact, molecular data alone cannot resolve this issue at the end without experimental infections using known parental genotypes. Regarding incomplete lineage sorting, use of additional marker sequences is highly recommended. In the context of the present project, it was also aimed to evaluate proteomic (MALDI-TOF MS) data for cyathostomin species^25^ and this limited the number of DNA sequences that could reliably amplified per specimen. Therefore, it was decided to focus on the two markers that showed the most robust amplification and the best chance to obtain both, phylogenetic signal and barcoding sequences. Another problem that should be mentioned is the fact that frequently nuclear pseudogenes of mitochondrial genes (numts) such as COI do occur. Data regarding the occurrence of numts in nematode genomes are scarce but they have been described for the filaroid species *Manzonella ozzardi*^38^. However, it can be excluded that such pseudogenes had a relevant influence on the data. Their copy number (single copy) is considerably smaller than that of mitochondrial genes. Moreover, they are very likely to contain missense mutations^39^. In very few cases when COI sequences did not contain complete open reading frames, another clone was picked and sequenced. It was not possible in the context of the project to determine whether such rare sequences were caused by PCR or cloning artefacts or by picking a bacterial cloning with an insert representing a numt.

In conclusion, each of the two marker genes analysed has its advantages and disadvantages. By using both genes simultaneously in the present analysis, it was possible to obtain a better understanding of the genetic differences of each species. The hereby generated evidence further confirms the hypotheses of cryptic species complexes in *Cys. minutus* and *Cys. calicatus*. This study does not support the current taxonomical classification for some genera based on either analyzed genes. It is also noteworthy that there was no obvious differentiation in genotypes associated with equine host species and geographic regions (Ukraine vs. Germany).

In the long term, the project aims to obtain data allowing successful metabarcoding of cyathostomin species in particular from fecal samples to identify resistant species, correlate clinical signs with parasite species patterns of cyathostomine infections and understand the ecology of the different cyathostomine species including host preference (e.g. foals versus adults). For this purpose, a database with reliable taxonomic information containing unambiguous barcoding sequences is required. However, a robust knowledge about the phylogenetic relationship of the species will also be required since more closely related species might share more parasitological features, clinical consequences and ecological niches than more distantly related species. Looking at the combinations of nuclear and mitochondrial markers also helped to determine whether mitochondrial genotypes might represent potential cryptic species (fixed combinations of nuclear and mitochondrial haplotypes) or not (free mixing of nuclear and mitochondrial haplotypes). Stepwise addition of further species to the database containing taxonomic, phylogenetic and barcoding information is ongoing.

## Materials and methods

### Specimen collection

Adult cyathostomin specimens were collected from eight domestic horses *Equus ferus caballus* from Germany during necropsy. All animal procedures and protocols were conducted in agreement with European (directive 2010/63/EU) and national (Tierschutzgesetzt) legislation and were approved by the LAGeSo Berlin (file number A 0237/14). Cyathostomin adults were also collected from fecal samples post-treatment with 0.2% aversectin C (“Univerm”, 0.2% aversectin C, PharmBioMed, Russia) of one individual each of five equine host species residing at Askania-Nova Biosphere reserve, Ukraine, i.e. *E. ferus caballus*, *E. ferus przewalskii*, *E. africanus asinus*, *E. hemionus kulan* and *E. quagga burchelli*. All collected specimens were washed in distilled water and fixed in 70% ethanol. Individual worms were identified to species level based on their morphological characteristics^1^. Only specimens identified as *Cyc. nassatus*, *Cor. labiatus*, *Cor. coronatus*, *Cyc. longibursatus, Cys. minutus* and *Cys. calicatus* were included in the remainder of study. For each individual equine host, up to ten adult individuals (usually five males and five females) of each of these cyathostomin species were selected for downstream processing.

### DNA isolation, PCR and sequencing

After DNA extraction of individual cyathostomin specimens with the NucleoSpin Tissue XS Kit (Macherey–Nagel, Düren, Germany), PCRs targeting the internal transcribed spacer 2 (ITS-2)^40^ and a partial cytochrome c oxidase I (COI)^26^ were conducted using a high-fidelity DNA polymerase^25^. PCR products were cloned into the pSC-Bamp/kan vector using the StrataClone Blunt PCR Cloning Kit (Agilent Technologies, Waldbronn, Germany), and one clone with insert per PCR fragment of each individual worm was sequenced by LGC Genomics (Berlin).

### Phylogenetic analyses

Sequences were manually edited using MEGA7 software^41^ by removing the vector and primers. ITS-2 sequences were aligned using MAFFT^42^ with default parameters and -G-INS-i as iterative refinement method. COI sequences aligned unambiguously without gaps. To investigate whether substitution saturation occurred in the ITS-2, the COI codon positions 1 and 2 as well as COI codon position 3, frequencies of transitions and transversions were plotted against the Juces-Cantor genetic distance for each of these alignments using DAMBE 7.0.35^43^. In addition, tests for substitution saturation were conducted using the test described by Xia et al.^44,45^. Maximum likelihood phylogenetic analyses were conducted using the IQ-TREE web server^46^. The ModelFinder option of IQ-TREE^47^ was set to auto-determination of the best model, and models with FreeRate heterogeneity were included. Ultrafast bootstrapping (1000 bootstrapped alignments)^48^ and the Shimodaira-Hasegawa approximate likelihood ratio test (1000 replicates) were chosen to obtain node support statistics. For the protein coding COI sequences, separate models were fitted for codon positions 1 and 2 versus codon position 3^49,50^. A combined ITS-2/COI tree was calculated using three partitions (ITS-2, COI codon position 1&2, COI codon position 3). The trees were visualized and edited using FigTree software^51^. *Cylicostephanus minutus* was chosen to root the trees since it was at the most basal position of the six included species in a previously published analysis^52^.

### Sequence identities

Identities between sequences were calculated for ITS-2 and COI using the R package ape version 5.2^53^ in R software version 3.5.0^54^. R software was accessed via R Studio version 1.1.463. For this purpose, the same alignments were used as for calculation of the respective phylogenetic trees without differentiating between codon positions were used. The resulting matrix was manually converted to columns of identity between groups of sequences, according to the clusters observed in the trees. Box plots comparing the identity between groups were created with GraphPad Prism 8 software. Kruskal–Wallis tests followed by a Dunn’s multiple comparison tests were used to identify significant differences in identity between different groups considering *p* < 0.05 as significant.

## Supplementary Information

Supplementary Information
