# Supplementary material for: Genetic variability, cryptic species and phylogenetic relationship of six cyathostomin species based on mitochondrial and nuclear sequences
Source: Sci Rep. 2021 Apr 15;11:8245. doi: 10.1038/s41598-021-87500-8 (PMC8050097; doi:10.1038/s41598-021-87500-8)
Supplement: Supplementary file 1 — Supplementary Information [file 41598_2021_87500_MOESM1_ESM.pdf]

Genetic variability, cryptic species and phylogenetic relationship of six cyathostomin species based on mitochondrial and nuclear sequences

Mariana Louro[1,2], Tetiana A. Kuzmina[3], Christina M. Bredtmann[1], Irina Diekmann[1], Luís M. Madeira de Carvalho[2], Georg von Samson-Himmelstjerna[1], Jürgen Krücken[1,*]

[1] Institute for Parasitology and Tropical Veterinary Medicine, Department of Veterinary Medicine, Freie Universität Berlin, Germany

[2] CIISA – Centre for Interdisciplinary Research in Animal Health, Faculty of Veterinary Medicine, University of Lisbon, Portugal

[3] I. I. Schmalhausen Institute of Zoology, National Academy of Sciences of Ukraine, Kyiv, Ukraine

[*] email: juergen.kruecken@fu-berlin.de

Table S1
Description of parasite specimens and accession numbers

Species	Hosts	Origin	Sex[a]	n[b]	Code	ITS-2 No.	COI No.
Cylicostephanus calicatus	Donkey	Ukraine	f	01	CAL_D_F01	MW366998	MH247954
				02	CAL_D_F02	MW367005	MH247956
				03	CAL_D_F03	MW366998	MH247928
				04	CAL_D_F04	MW366998	MH247956
				05	CAL_D_F05	MW367006	MH247929
				06	CAL_D_F06	MW367008	MH247930
				07	CAL_D_F07	MW367009	MH247931
			m	01	CAL_D_M01	MW367002	MH247932
				02	CAL_D_M02	MW367003	MH247933
				03	CAL_D_M03	MW366998	MH247934
	Horse	Germany	f	01	CAL_G_F01	MW367009	MH247935
				02	CAL_G_F02	MW367017	-
				03	CAL_G_F03	MW367007	MH247937
				04	CAL_G_F04	MW367004	MH247940
				05	CAL_G_F05	MW367018	MH247938
				06	CAL_G_F06	MW366998	MH247939
				07	CAL_G_F07	MW367025	-
				08	CAL_G_F08	MW366998	MH247941
				09	CAL_G_F09	MW367020	MH247942
				10	CAL_G_F10	MW367019	MH247943
				11	CAL_G_F11	MW367003	MH247944
				12	CAL_G_F12	MW367024	MH247945
				13	CAL_G_F13	MW366998	MH247946
			m	01	CAL_G_M01	MW366998	MH247947
				02	CAL_G_M02	MW367010	MH247948

				03	CAL_G_M03	MW367002	MH247949
				04	CAL_G_M04	MW367002	MH247950
				05	CAL_G_M05	MW367022	MH247951
				06	CAL_G_M06	MW367020	MH247952
				07	CAL_G_M07	MW367019	MH247953
		Ukraine	f	01	CAL_H_F01	MW367002	MH247954
				02	CAL_H_F02	MW366999	MH247955
				03	CAL_H_F03	MW366998	MH247956
				04	CAL_H_F04	MW367003	MH247956
				05	CAL_H_F05	MW367002	MH247957
			m	01	CAL_H_M01	MW366998	MH247958
				02	CAL_H_M02	MW367002	MH247959
				03	CAL_H_M03	MW367000	MH247960
				04	CAL_H_M04	MW366998	MH247961
				05	CAL_H_M05	MW367010	MH247962
	Kulan	Ukraine	f	01	CAL_K_F01	MW367011	-
				02	CAL_K_F02	MW367013	-
				03	CAL_K_F03	MW367021	-
				04	CAL_K_F04	MW367012	-
				05	CAL_K_F05	MW366998	MH247965
				06	CAL_K_F06	MW367018	MH247966
				07	CAL_K_F07	MW367023	-
				08	CAL_K_F08	MW367009	MH247967
			m	01	CAL_K_M01	MW367015	-
				02	CAL_K_M02	MW367018	MH247969
	Przewalski's	Ukraine	f	01	CAL_P_F01	MW367026	MH247970
				02	CAL_P_F02	MW367011	-
				03	CAL_P_F03	MW367011	MH247971

				04	CAL_P_F04	MW367018	MH247972
				05	CAL_P_F05	MW367018	MH247973
				06	CAL_P_F06	MW367022	MH247974
				07	CAL_P_F07	MW367020	MH247975
				08	CAL_P_F08	MW367016	-
				09	CAL_P_F09	MW367012	MH247977
			m	01	CAL_P_M01	MW367014	MH247978
	Zebra	Ukraine	f	01	CAL_Z_F01	MW366998	MH247979
				02	CAL_Z_F02	MW367002	MH247980
			m	01	CAL_Z_M01	MW367001	MH247981
Coronocyclus coronatus	Donkey	Ukraine	f	01	COR_D_F01	MW367030	MW363695
				02	COR_D_F02	MW367030	MW363696
				03	COR_D_F03	MW367030	MW363697
				04	COR_D_F04	MW367030	MW363698
				05	COR_D_F05	MW367039	MW363699
			m	01	COR_D_M01	MW367030	MW363700
				02	COR_D_M02	MW367030	MW363747
				03	COR_D_M03	MW367030	MW363701
				04	COR_D_M04	MW367030	MW363739
				05	COR_D_M05	MW367030	MW363702
	Horse	Germany	f	01	COR_G_F01	MW367031	MW363703
				02	COR_G_F02	MW367030	MW363704
				03	COR_G_F03	MW367037	MW363705
				04	COR_G_F04	MW367030	MW363706
				05	COR_G_F05	MW367030	MW363707
			m	01	COR_G_M01	MW367030	MW363708
				02	COR_G_M02	MW367030	MW363709
				03	COR_G_M03	MW367030	MW363710

				04	COR_G_M04	MW367030	MW363711
				05	COR_G_M05	MW367030	MW363712
		Ukraine	f	01	COR_H_F01	MW367032	MW363713
				02	COR_H_F02	MW367030	-
				03	COR_H_F03	MW367030	MW363714
				04	COR_H_F04	MW367030	MW363715
				05	COR_H_F05	MW367030	MW363716
			m	01	COR_H_M01	MW367030	MW363717
				02	COR_H_M02	MW367030	MW363718
				03	COR_H_M03	MW367030	MW363719
				04	COR_H_M04	MW367030	MW363720
				05	COR_H_M05	MW367030	MW363721
	Kulan	Ukraine	f	01	COR_K_F01	MW367030	MW363722
				02	COR_K_F02	MW367030	MW363723
				03	COR_K_F03	MW367030	MW363724
				04	COR_K_F04	MW367030	MW363725
				05	COR_K_F05	MW367030	MW363726
				06	COR_K_F06	MW367030	MW363727
				07	COR_K_F07	MW367030	MW363728
				08	COR_K_F08	MW367030	MW363729
				09	COR_K_F09	MW367030	MW363730
				10	COR_K_F10	MW367030	MW363731
	Przewalski's	Ukraine	f	01	COR_P_F01	MW367030	MW363732
				02	COR_P_F02	MW367030	MW363733
				03	COR_P_F03	MW367033	MW363734
				04	COR_P_F04	MW367030	MW363735
				05	COR_P_F05	MW367030	MW363736
			m	01	COR_P_M01	MW367038	MW363737

				02	COR_P_M02	MW367030	MW363738
				03	COR_P_M03	MW367030	MW363739
				04	COR_P_M04	MW367034	MW363740
				05	COR_P_M05	MW367034	MW363741
	Zebra	Ukraine	f	01	COR_Z_F01	MW367030	MW363742
				02	COR_Z_F02	MW367035	MW363743
				03	COR_Z_F03	MW367030	MW363744
				04	COR_Z_F04	MW367030	MW363744
				05	COR_Z_F05	MW367030	MW363745
			m	01	COR_Z_M01	MW367036	MW363746
				02	COR_Z_M02	MW367030	MW363747
				03	COR_Z_M03	MW367030	MW363748
				04	COR_Z_M04	MW367030	MW363749
				05	COR_Z_M05	MW367030	MW363750
Coronocyclus labiatus	Donkey	Ukraine	f	01	LAB_D_F01	MW243599	MW222992
				02	LAB_D_F02	MW243604	MW222997
				03	LAB_D_F03	MW243599	MW222956
				04	LAB_D_F04	MW243599	MW222957
				05	LAB_D_F05	MW243599	MW222980
			m	01	LAB_D_M01	MW243599	MW222997
				02	LAB_D_M02	MW243605	MW222997
				03	LAB_D_M03	MW243589	MW222993
				04	LAB_D_M04	MW243604	MW222980
				05	LAB_D_M05	MW243599	MW222958
	Horse	Germany	f	01	LAB_G_F01	MW243599	MW222959
				02	LAB_G_F02	MW243590	MW222994
				03	LAB_G_F03	MW243589	MW222960
				04	LAB_G_F04	MW243599	MW222961

				05	LAB_G_F05	MW243599	MW222995
			m	01	LAB_G_M01	MW243599	MW222962
				02	LAB_G_M02	MW243591	MW222963
				03	LAB_G_M03	MW243589	MW222999
				04	LAB_G_M04	MW243589	MW222964
				05	LAB_G_M05	MW243599	MW222965
		Ukraine	f	01	LAB_H_F01	MW243589	MW222984
				02	LAB_H_F02	MW243599	MW222966
				03	LAB_H_F03	MW243599	MW222476
				04	LAB_H_F04	MW243599	MW222988
				05	LAB_H_F05	MW243599	MW222984
			m	01	LAB_H_M01	MW243589	MW222967
				02	LAB_H_M02	MW243601	MW222968
				03	LAB_H_M03	MW243592	MW222996
				04	LAB_H_M04	MW243605	MW222999
				05	LAB_H_M05	MW243589	MW222997
	Kulan	Ukraine	f	01	LAB_K_F01	MW243599	MW222969
				02	LAB_K_F02	MW243589	MW222970
				03	LAB_K_F03	MW222414	MW222998
				04	LAB_K_F04	MW243599	MW222980
				05	LAB_K_F05	MW243599	MW222971
			m	01	LAB_K_M01	MW243600	MW222971
				02	LAB_K_M02	MW243599	MW222972
				03	LAB_K_M03	MW243589	MW222973
				04	LAB_K_M04	MW243589	MW222999
				05	LAB_K_M05	MW243599	MW223000
	Przewalski's	Ukraine	f	01	LAB_P_F01	MW243601	MW223001
				02	LAB_P_F02	MW243599	MW222974

				03	LAB_P_F03	MW243602	MW222975
				04	LAB_P_F04	MW243599	MW222976
				05	LAB_P_F05	MW243599	MW222977
			m	01	LAB_P_M01	MW243608	MW222978
				02	LAB_P_M02	MW243606	MW222979
				03	LAB_P_M03	MW243607	MW223002
				04	LAB_P_M04	MW243599	MW222980
				05	LAB_P_M05	MW243599	MW222980
	Zebra	Ukraine	f	01	LAB_Z_F01	MW243599	MW222981
				02	LAB_Z_F02	MW243599	MW222982
				03	LAB_Z_F03	MW243599	MW222983
				04	LAB_Z_F04	MW243599	MW222985
				05	LAB_Z_F05	MW243599	MW222984
				06	LAB_Z_F06	MW243599	MW223003
			m	01	LAB_Z_M01	MW243599	MW222985
				02	LAB_Z_M02	MW243599	MW222986
				03	LAB_Z_M03	MW243603	MW222987
				04	LAB_Z_M04	MW243589	MW222988
Cylicostephanus longibursatus	Donkey	Ukraine	f	01	LON_D_F01	MW282922	MW256719
			m	01	LON_D_M01	MW282929	MW256720
				02	LON_D_M02	MW282925	MW256721
				03	LON_D_M03	MW282931	MW256722
	Horse	Germany	f	01	LON_G_F01	MH483930	MH247982
				02	LON_G_F02	MH483931	MH247983
				03	LON_G_F03	MH483932	MH247984
				04	LON_G_F04	MH483943	MH247985
				05	LON_G_F05	MH483938	MH247986
			m	01	LON_G_M01	MH483933	MH247987

				02	LON_G_M02	MH483942	MH247988
				03	LON_G_M03	MH483934	MH247989
				04	LON_G_M04	MH483935	MH247990
				05	LON_G_M05	MH483936	MH247991
		Ukraine	f	01	LON_H_F01	MH483937	MH247992
				02	LON_H_F02	MH483938	MH247993
				03	LON_H_F03	MH483939	MH247994
				04	LON_H_F04	MH483940	MH247995
				05	LON_H_F05	MH483941	MH247996
			m	01	LON_H_M01	MH483942	MH247997
				02	LON_H_M02	MH483943	MH247998
				03	LON_H_M03	MH483944	MH247999
				04	LON_H_M04	MH483945	MH248000
				05	LON_H_M05	MH483945	MH248001
	Kulan	Ukraine	f	01	LON_K_F01	MW282927	MW256723
				02	LON_K_F02	MW282939	MW256724
				03	LON_K_F03	MW282938	MW256725
				04	LON_K_F04	MW282929	MW256726
				05	LON_K_F05	MW282930	MW256727
			m	01	LON_K_M01	MW282928	MW256728
				02	LON_K_M02	MW282938	MW256729
				03	LON_K_M03	MW282929	MW256730
				04	LON_K_M04	MW282937	MW256731
				05	LON_K_M05	MW282923	MW256732
	Przewalski's	Ukraine	f	01	LON_P_F01	MW282938	MW256733
				02	LON_P_F02	MW282935	MW256734
				03	LON_P_F03	MW282923	MW256735
				04	LON_P_F04	MW282923	MW256736

				05	LON_P_F05	MW282923	MW256738
			m	01	LON_P_M01	MW282941	MW256737
				02	LON_P_M02	MW282936	MW256738
				03	LON_P_M03	MW282932	MW256739
				04	LON_P_M04	MW282936	MW256740
				05	LON_P_M05	MW282933	MW256741
	Zebra	Ukraine	f	01	LON_Z_F01	MW282924	MW256742
				02	LON_Z_F02	MW282929	MW256743
				03	LON_Z_F03	MW282934	MW256744
				04	LON_Z_F04	MW282926	MW256745
				05	LON_Z_F05	MW282940	MW256746
			m	01	LON_Z_M01	MW282936	MW256747
				02	LON_Z_M02	MW282938	MW256748
				03	LON_Z_M03	MW282923	MW256749
				04	LON_Z_M04	MW282929	MW256750
				05	LON_Z_M05	MW282923	MW256751
Cylicostephanus minutus	Donkey	Ukraine	f	01	MIN_D_F01	MW282942	MW256763
				02	MIN_D_F02	MW282942	MW256764
				03	MIN_D_F03	MW282943	MW256765
				04	MIN_D_F04	MW282951	MW256766
				05	MIN_D_F05	MW282943	MW256770
			m	01	MIN_D_M01	MW282942	MW256767
				02	MIN_D_M02	MW282946	MW256768
				03	MIN_D_M03	MW282948	MW256769
				04	MIN_D_M04	MW282946	MW256770
				05	MIN_D_M05	MW282946	MW256771
	Horse	Germany	f	01	MIN_G_F01	MH487672	MH460777
				02	MIN_G_F02	MH487659	MH460767

			03	MIN_G_F03	MH487658	MH460768
			04	MIN_G_F04	MH487658	MH460769
			05	MIN_G_F05	MH487657	MH460770
		m	01	MIN_G_M01	MH487658	MH460771
			02	MIN_G_M02	MH487668	MH460778
			03	MIN_G_M03	MH487672	MH460779
			04	MIN_G_M04	MH487667	MH460780
			05	MIN_G_M05	MH487658	MH460772
	Ukraine	f	01	MIN_H_F01	MH487668	MH460782
			02	MIN_H_F02	MH487669	MH460783
			03	MIN_H_F03	MH487671	MH460784
			04	MIN_H_F04	MH487671	MH460785
			05	MIN_H_F05	MH487670	MH460786
		m	01	MIN_H_M01	MH487671	MH460787
			02	MIN_H_M02	MH487672	MH460788
Kulan	Ukraine	f	01	MIN_K_F01	MW282952	MW256772
			02	MIN_K_F02	MW282950	MW256773
			03	MIN_K_F03	MW282952	MW256774
			04	MIN_K_F04	MW282942	MW256775
			05	MIN_K_F05	MW282952	MW256776
		m	01	MIN_K_M01	MW282946	MW256777
			02	MIN_K_M02	MW282952	MW256778
			03	MIN_K_M03	MW282952	MW256779
			04	MIN_K_M04	MW282952	MW256780
			05	MIN_K_M05	MW282953	MW256781
Przewalski's	Ukraine	f	01	MIN_P_F01	MW282948	MW256782
			02	MIN_P_F02	MW282944	MW256783
			03	MIN_P_F03	MW282946	MW256784

				04	MIN_P_F04	MW282945	MW256785
				05	MIN_P_F05	MW282942	MW256786
			m	01	MIN_P_M01	MW282947	MW256787
				02	MIN_P_M02	MW282942	MW256788
				03	MIN_P_M03	MW282946	MW256789
				04	MIN_P_M04	MW282944	MW256790
				05	MIN_P_M05	MW282942	MW256791
	Zebra	Ukraine	f	01	MIN_Z_F01	MW282946	MW256792
				02	MIN_Z_F02	MW282942	MW256793
				03	MIN_Z_F03	MW282952	MW256794
			m	01	MIN_Z_M01	MW282944	MW256795
				02	MIN_Z_M02	MW282949	MW256796
Cylicocyclus nassatus	Donkey	Ukraine	f	01	NAS_D_F01	MW208845	MW203127
				02	NAS_D_F02	MW208845	MW199167
				03	NAS_D_F03	MW208845	MW203128
				04	NAS_D_F04	MW208845	MW203129
				05	NAS_D_F05	MW208845	MW203130
			m	01	NAS_D_M01	MW208849	MW203131
				02	NAS_D_M02	MW208845	MW203132
				03	NAS_D_M03	MW208845	MW203133
				04	NAS_D_M04	MW208845	MW203134
				05	NAS_D_M05	MW208851	MW203135
	Horse	Germany	f	01	NAS_G_F01	MW208846	MW203136
				02	NAS_G_F02	MW208852	MW203138
				03	NAS_G_F03	MW208855	MW203139
				04	NAS_G_F04	MW208845	MW203140
				05	NAS_G_F05	MW208847	MW203141
			m	01	NAS_G_M01	MW208845	MW203137

				02	NAS_G_M02	MW208845	MW203138
				03	NAS_G_M03	MW208845	MW203139
				04	NAS_G_M04	MW208854	MW203140
				05	NAS_G_M05	MW208845	MW203141
		Ukraine	f	01	NAS_H_F01	MW208850	MW203142
				02	NAS_H_F02	MW208857	MW203143
				03	NAS_H_F03	MW208845	MW203144
				04	NAS_H_F04	MW208845	MW203145
				05	NAS_H_F05	MW208853	MW203146
			m	01	NAS_H_M01	MW208845	MW203150
				02	NAS_H_M02	MW208856	-
				03	NAS_H_M03	MW208845	MW203147
				04	NAS_H_M04	MW211119	MW203148
				05	NAS_H_M05	MW208851	MW203149
	Kulan	Ukraine	f	01	NAS_K_F01	MW208845	MW203150
				02	NAS_K_F02	MW208845	MW203151
				03	NAS_K_F03	MW208845	MW203152
				04	NAS_K_F04	MW208845	MW203153
				05	NAS_K_F05	MW208845	MW203154
			m	01	NAS_K_M01	MW208845	MW203155
				02	NAS_K_M02	MW208845	MW203156
				03	NAS_K_M03	MW208845	MW203157
				04	NAS_K_M04	MW208850	MW203158
				05	NAS_K_M05	MW208850	MW203159
	Przewalski's	Ukraine	f	01	NAS_P_F01	MW198060	MW197162
				02	NAS_P_F02	MW208845	MW203160
				03	NAS_P_F03	MW208845	MW203161
				04	NAS_P_F04	MW208848	MW203162

			05	NAS_P_F05	MW208851	MW203163
		m	01	NAS_P_M01	MW208851	MW203164
			02	NAS_P_M02	MW208851	MW203165
			03	NAS_P_M03	MW208845	MW203166
			04	NAS_P_M04	MW208845	MW203167
			05	NAS_P_M05	MW208845	MW203168
Zebra	Ukraine	f	01	NAS_Z_F01	MW208853	MW203169
			02	NAS_Z_F02	MW208853	MW203170
			03	NAS_Z_F03	MW208845	MW203171
			04	NAS_Z_F04	MW208845	MW203172
			05	NAS_Z_F05	MW208845	MW203173
		m	01	NAS_Z_M01	MW208845	MW203174
			02	NAS_Z_M02	MW208845	MW203175
			03	NAS_Z_M03	MW208845	MW203176
			04	NAS_Z_M04	MW208850	MW203177
			05	NAS_Z_M05	MW208845	MW203178

[a] f, female; m, male

[b] Consecutive number starting with one for every combination of host and sex.

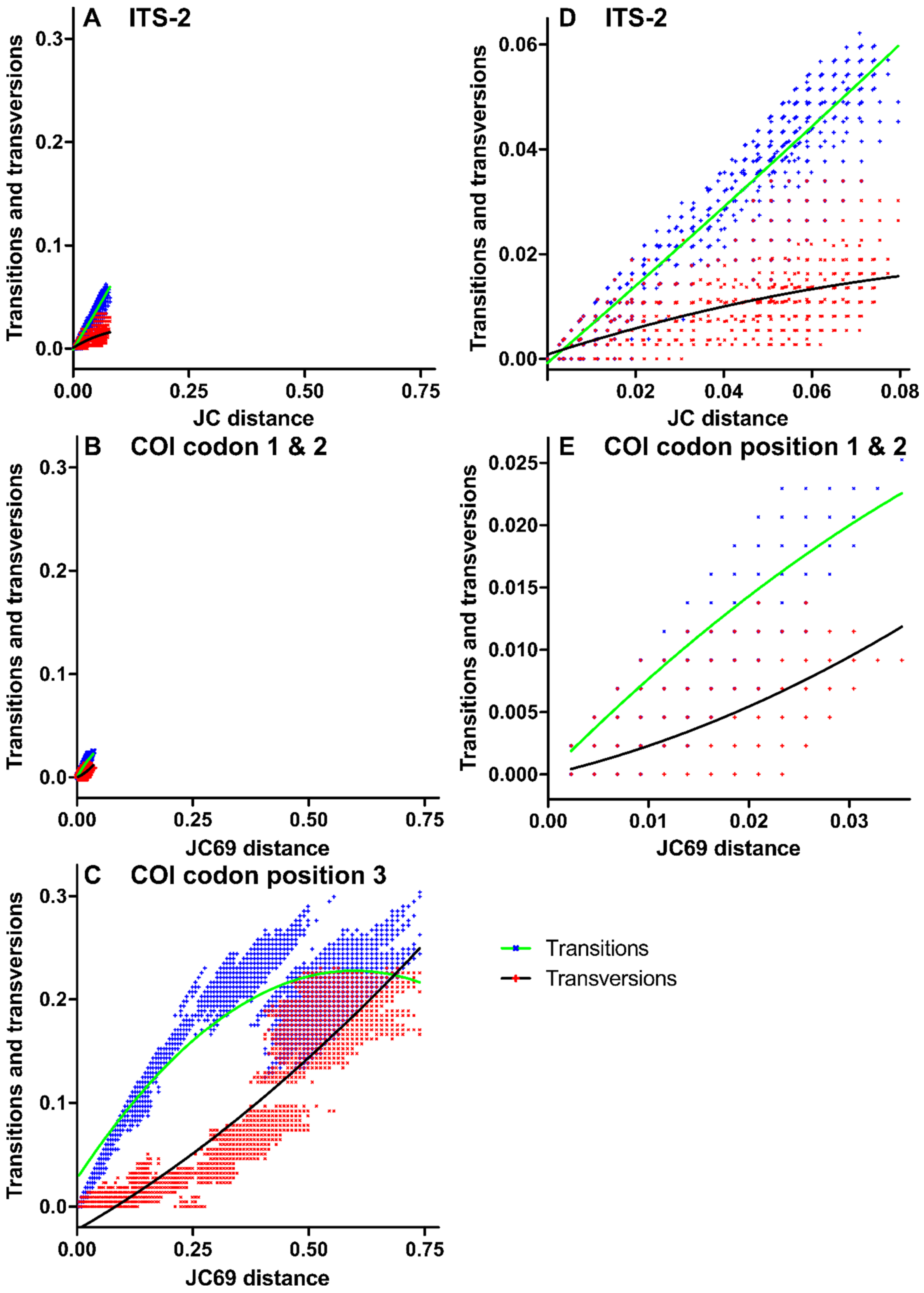

Figure S1. Frequency of transitions and transversions are plotted versus the Jukes-Cantor (JC) genetic distance. On the left (A-C), all plots have identically scaled axes to show the much higher frequencies of transitions and transversions in the third codon position of the cytochrome oxidase I (COI) gene compared to first and second codon position of this gene and the internal transcribed spacer-2 (ITS-2). On the right (D, E), plots for ITS-2 and COI are rescaled to optimize data visibility. Trend lines were calculated as quadratic regressions.

Table S2

Results of the substitution saturation test according to Xi et al[44,45].

Alignment	Number OTUs[a]	Iss[b]	Iss.cSym[c]	T value[d]	df[e]	p value[f]	Iss.cAsym[g]	t value[h]	df[i]	p value[j]
ITS-2										
Frequency invariante sites 0.19										
	4	0.284	0.786	16.707	298	0	0.756	15.707	298	0
	8	0.338	0.739	9.747	298	0	0.629	7.062	298	0
	16	0.353	0.696	7.278	298	0	0.487	2.842	298	0.0048
	32	0.408	0.688	4.935	298	0	0.358	0.88	298	0.3796
COI codon position 1 and 2										
Frequency invariante sites 0.38										
	4	0.026	0.791	77.36	271	0	0.758	74.04	271	0
	8	0.026	0.745	70.001	271	0	0.634	59.143	271	0
	16	0.028	0.709	66.507	271	0	0.5	46.074	271	0
	32	0.029	0.695	65.989	271	0	0.367	33.539	271	0
COI codon position 3										
Frequency invariante sites 0.03										
	4	0.486	0.777	8.267	216	0	0.764	7.902	216	0
	8	0.474	0.733	7.188	216	0	0.633	4.41	216	0
	16	0.47	0.65	4.922	216	0	0.457	0.36	216	0.7192
	32	0.469	0.687	5.923	216	0	0.368	2.777	216	0.006

[a]Number of operational taxonomic units randomly samples from the alignment to be used in the test. Test was conducted with 60 replicates.

[b]Index of substitution saturation.

[c]Critical index of substitution saturation for a symmetric tree.

[d]t-value for two-tailed t test of Iss vs. Iss.cSym.

[e]Dregrees of freedom for two-tailed t test of Iss vs. Iss.cSym.

[f]p value for two-tailed t test of Iss vs. Iss.cSym

[g]Critical index of substitution saturation for the most asymmetric tree.

[h]t-value for two-tailed t test of Iss vs. Iss.cAsym.

[i]Dregrees of freedom for two-tailed t test of Iss vs. Iss.cAsym.

[j]p value for two-tailed t test of Iss vs. Iss.cAsym

Table S3

Chosen substitution models and parameters

					Rate parameters						Base frequencies			
Partition	Model	p_{invar}	Γ shape α	Relative categories & rate (Proportion)	A-C	A-G	A-T	C-G	C-T	G-T	A	C	G	T
ITS-2[a]	K2P+G4		0.468		1.00000	5.02637	1.00000	1.00000	5.02637	1.00000	A: 0.250	A: 0.250	A: 0.250	A: 0.250
COI 1 & 2[b]	TIM3+F+R2			1. 0.3299 (0.924)<br>2. 9.152 (0.07596)	4.8405	5.4176	1.0000	4.8405	23.7904	1.0000	0.227	0.1646	0.2182	0.3903
COI position 3[c]	TIM3+F+I+G4	0.1022	2.119	0. 0 (0.1022)<br>1. 0.3421 (0.2244)<br>2. 0.7431 (0.2244)<br>3. 1.195 (0.2244)<br>4. 2.176 (0.2244)	2.5633	23.8269	1.0000	2.5633	77.8017	1.0000	0.2715	0.04207	0.2169	0.4695

Data S1. ITS-2 phylogenetic tree in Nexus format.

#NEXUS

begin trees;

tree tree_1 = [&R]
((((((((((((((((((("MIN_D_F01":0,"MIN_D_M01":0):0,"MIN_H_M01":0):0,"MIN_Z_F02":0):0,"MIN_P_F0
5":0):0,"MIN_P_M05":0):0,"MIN_H_F04":0):0,"MIN_H_F03":0):0,"MIN_K_F04":0):0,"MIN_P_M02":0)
:0.000002,"MIN_D_F02":0.000002)[&"SH/boot"="0/43"]:0.000002,(((((("MIN_G_M02":0,"MIN_P_F0
2":0):0,"MIN_P_M04":0):0,"MIN_Z_M01":0):0.000002,"MIN_H_F01":0.000002)[&"SH/boot"="0/62"]
:0.000002,"MIN_D_F04":0.013652)[&"SH/boot"="85.9/90"]:0.004444,"MIN_P_F04":0.004426)[&"SH
/boot"="0/11"]:0.000002)[&"SH/boot"="0/25"]:0.000002,("MIN_D_F03":0.000002,"MIN_D_F05":0.0
00002)[&"SH/boot"="84.5/99"]:0.004407)[&"SH/boot"="84.8/90"]:0.004406,"MIN_K_F02":0.009008
)[&"SH/boot"="0/52"]:0.000003,"MIN_H_F02":0.000003)[&"SH/boot"="85.7/92"]:0.004446,((((("MI
N_D_M03":0,"MIN_G_M03":0):0,"MIN_H_M02":0):0,"MIN_P_F01":0):0.000002,"MIN_G_F01":0.000
002)[&"SH/boot"="0/48"]:0.000002,("MIN_G_M04":0.004407,"MIN_Z_M02":0.0089)[&"SH/boot"="
0/41"]:0.000002)[&"SH/boot"="0/25"]:0.000003)[&"SH/boot"="64.5/83"]:0.004444,(((((((("MIN_D_
M02":0,"MIN_P_F03":0):0,"MIN_P_M03":0):0,"MIN_K_M01":0):0,"MIN_H_F05":0):0,"MIN_D_M05":
0):0,"MIN_Z_F01":0):0.000002,"MIN_D_M04":0.000002)[&"SH/boot"="0/50"]:0.000002,"MIN_P_M0
1":0.004426)[&"SH/boot"="0/66"]:0.000002)[&"SH/boot"="89.6/92"]:0.016874,(((((((((((("MIN_G_F0
3":0,"MIN_K_M02":0):0,"MIN_G_M01":0):0,"MIN_K_F03":0):0,"MIN_K_F01":0):0,"MIN_Z_F03":0):0,
"MIN_G_M05":0):0,"MIN_K_M04":0):0,"MIN_K_M03":0):0,"MIN_K_F05":0):0.000002,"MIN_G_F04":
0.000002)[&"SH/boot"="0/65"]:0.000002,"MIN_K_M05":0.004407)[&"SH/boot"="76.5/98"]:0.00434
5,"MIN_G_F05":0.009029)[&"SH/boot"="96/99"]:0.023218)[&"SH/boot"="80.3/91"]:0.010909,"MIN
_G_F02":0.038794)[&"SH/boot"="89/92"]:0.007123,(((((((((((((("LON_D_F01":0.002749,("LON_Z_F01
":0.002749,"LON_H_F01":0.005541)[&"SH/boot"="0/23"]:0.000002)[&"SH/boot"="0/5"]:0.000002,"L
ON_H_M01":0.000002)[&"SH/boot"="0/2"]:0.000002,((((((("LON_G_M02":0,"LON_P_F04":0):0,"LON
_P_F03":0):0,"LON_P_F05":0):0,"LON_Z_M05":0):0,"LON_H_M03":0):0,"LON_Z_M03":0):0,"LON_K_
M05":0):0.000002)[&"SH/boot"="0/6"]:0.000002,("LON_H_F04":0.002763,"LON_K_M01":0.005541)[
&"SH/boot"="0/10"]:0.000002)[&"SH/boot"="91.3/68"]:0.002762,(("LON_D_M02":0.005516,(("LON_
G_M04":0.000002,"LON_Z_F04":0.000002)[&"SH/boot"="0/64"]:0.000002,"LON_K_F01":0.002748)[
&"SH/boot"="82.6/82"]:0.002749)[&"SH/boot"="0/26"]:0.000002,"LON_G_F03":0.000002)[&"SH/bo
ot"="0/20"]:0.000003)[&"SH/boot"="0/90"]:0.000002,(((((((((("LON_D_M01":0,"LON_K_F04":0):0,"L
ON_Z_M04":0):0,"LON_Z_F02":0):0,"LON_K_M03":0):0.000002,"LON_G_M05":0.000002)[&"SH/boot
"="0/56"]:0.000002,"LON_K_F05":0.002749)[&"SH/boot"="85.8/39"]:0.002749,((("LON_H_M04":0,"L
ON_P_M05":0):0.000002,"LON_H_M05":0.000002)[&"SH/boot"="0/55"]:0.000002,"LON_Z_F03":0.0
02748)[&"SH/boot"="82.3/99"]:0.002749)[&"SH/boot"="0/8"]:0.000002,"LON_D_M03":0.000002)[&
"SH/boot"="0/21"]:0.000002,"LON_P_M01":0.008351)[&"SH/boot"="86.3/16"]:0.002749,"LON_P_M
03":0.000002)[&"SH/boot"="89.3/51"]:0.005542)[&"SH/boot"="36.1/31"]:0.002788,("LON_G_F02":0
.000002,(((((((("LON_G_F04":0,"LON_Z_M02":0):0,"LON_K_M02":0):0,"LON_K_F03":0):0,"LON_P_F0
1":0):0.000002,"LON_H_M02":0.000002)[&"SH/boot"="0/43"]:0.000002,("LON_K_F02":0.002748,("L
ON_G_F05":0.000002,"LON_H_F02":0.000002)[&"SH/boot"="84.6/100"]:0.002749)[&"SH/boot"="0/
17"]:0.000002)[&"SH/boot"="0/25"]:0.000002,("LON_G_M03":0.002748,"LON_Z_F05":0.000002)[&"
SH/boot"="83.7/100"]:0.002749)[&"SH/boot"="75.7/99"]:0.002768,"LON_G_M01":0.005575)[&"SH/
boot"="81.1/88"]:0.002745)[&"SH/boot"="81.7/79"]:0.002748)[&"SH/boot"="79/58"]:0.002755,"LO
N_P_F02":0.002748)[&"SH/boot"="0/10"]:0.000002,(("LON_P_M02":0,"LON_Z_M01":0):0.000002,"L
ON_P_M04":0.000002)[&"SH/boot"="0/20"]:0.000002)[&"SH/boot"="0/5"]:0.000003,"LON_H_F05":
0.002756)[&"SH/boot"="0/19"]:0.000002,"LON_G_F01":0.005531)[&"SH/boot"="0/31"]:0.000002,("
LON_H_F03":0.002749,"LON_K_M04":0.000002)[&"SH/boot"="73.7/99"]:0.005516)[&"SH/boot"="95

.1/100"]:0.018903,(((((("CAL_G_F05":0,"CAL_P_F04":0):0,"CAL_K_M02":0):0,"CAL_P_F05":0):0.000002,"CAL_K_F06":0.000002)[&"SH/boot"="0/42"]:0.000002,("CAL_K_F07":0.004092,"CAL_P_F01":0.012556)[&"SH/boot"="0/10"]:0.000002)[&"SH/boot"="0/8"]:0.000002,"CAL_G_F07":0.008266)[&"SH/boot"="85.1/29"]:0.004092,((("CAL_G_F10":0.000002,"CAL_G_M07":0.000002)[&"SH/boot"="0/31"]:0.000002,((("COR_D_F01":0,"COR_Z_F04":0):0,"COR_D_M01":0):0,"COR_G_M05":0):0,"COR_Z_F03":0):0,"COR_D_M05":0):0,"COR_K_F10":0):0,"COR_H_M05":0):0,"COR_P_F04":0):0,"COR_K_F05":0):0,"COR_Z_M05":0):0,"COR_D_F04":0):0,"COR_Z_F01":0):0,"COR_H_M02":0):0,"COR_G_F04":0):0,"COR_P_F02":0):0,"COR_P_M03":0):0,"COR_P_F01":0):0,"COR_H_F05":0):0,"COR_D_M03":0):0,"COR_G_M02":0):0,"COR_H_F02":0):0,"COR_Z_M03":0):0,"COR_Z_M02":0):0,"COR_D_F03":0):0,"COR_P_M02":0):0,"COR_H_F04":0):0,"COR_K_F04":0):0,"COR_H_M01":0):0,"COR_K_F02":0):0,"COR_K_F03":0):0,"COR_K_F08":0):0,"COR_P_F05":0):0,"COR_G_M04":0):0,"COR_H_M04":0):0,"COR_Z_M04":0):0,"COR_K_F06":0):0,"COR_D_M02":0):0,"COR_G_M01":0):0,"COR_H_M03":0):0,"COR_G_F02":0):0,"COR_K_F01":0):0,"COR_G_M03":0):0,"COR_K_F09":0):0,"COR_G_F05":0):0,"COR_Z_F05":0):0,"COR_K_F07":0):0,"COR_D_M04":0):0,"COR_H_F03":0):0.000002,((("COR_H_F01":0.000002,"COR_D_F05":0.008316)[&"SH/boot"="87/99"]:0.00412,"COR_Z_F02":0.00412)[&"SH/boot"="0/11"]:0.000003,("COR_P_M04":0.000002,"COR_P_M05":0.000002)[&"SH/boot"="89.9/100"]:0.004119)[&"SH/boot"="0/2"]:0.000003)[&"SH/boot"="0/1"]:0.000002,"COR_D_F02":0.000002)[&"SH/boot"="0/1"]:0.000002,(("COR_G_F01":0.004119,"COR_Z_M01":0.004119)[&"SH/boot"="0/12"]:0.000003,(("COR_P_F03":0.004119,"COR_P_M01":0.004147)[&"SH/boot"="0/3"]:0.000002,"COR_G_F03":0.004154)[&"SH/boot"="0/2"]:0.000002)[&"SH/boot"="0/1"]:0.000002)[&"SH/boot"="0/19"]:0.000002)[&"SH/boot"="0/12"]:0.000002,(((("CAL_G_F09":0,"CAL_P_F07":0):0.000002,("CAL_G_M06":0.000002,"CAL_G_F12":0.004108)[&"SH/boot"="0/18"]:0.000002)[&"SH/boot"="0/83"]:0.000002,"CAL_K_F03":0.004092)[&"SH/boot"="83.7/57"]:0.004091,("CAL_G_M05":0.000002,"CAL_P_F06":0.000002)[&"SH/boot"="85.2/77"]:0.004092)[&"SH/boot"="0/5"]:0.000002)[&"SH/boot"="0/2"]:0.000003)[&"SH/boot"="83.7/90"]:0.004149)[&"SH/boot"="0/47"]:0.000002,(("NAS_D_F01":0,"NAS_H_F04":0):0,"NAS_K_F02":0):0,"NAS_P_M05":0):0,"NAS_G_M02":0):0,"NAS_H_M03":0):0,"NAS_D_M02":0):0,"NAS_K_M03":0):0,"NAS_Z_F04":0):0,"NAS_K_F04":0):0,"NAS_Z_M02":0):0,"NAS_P_F03":0):0,"NAS_D_M03":0):0,"NAS_G_M01":0):0,"NAS_P_M03":0):0,"NAS_K_F05":0):0,"NAS_D_M04":0):0,"NAS_D_F04":0):0,"NAS_Z_M01":0):0,"NAS_H_F03":0):0,"NAS_K_M01":0):0,"NAS_P_M04":0):0,"NAS_K_F01":0):0,"NAS_Z_F03":0):0,"NAS_G_M05":0):0,"NAS_D_F05":0):0,"NAS_K_M02":0):0,"NAS_Z_M03":0):0,"NAS_Z_M05":0):0,"NAS_G_M03":0):0,"NAS_P_F02":0):0,"NAS_K_F03":0):0,"NAS_Z_F05":0):0,"NAS_D_F03":0):0,"NAS_H_M01":0):0,"NAS_G_F04":0):0.000002,((("NAS_G_F01":0.002748,(((((("NAS_D_M05":0,"NAS_P_M01":0):0,"NAS_P_F05":0):0,"NAS_P_M02":0):0.000002,"NAS_H_M05":0.000002)[&"SH/boot"="0/82"]:0.000002,"NAS_G_F02":0.002748)[&"SH/boot"="85.7/81"]:0.002748,(("NAS_H_F05":0,"NAS_Z_F02":0):0.000003,"NAS_Z_F01":0.000002)[&"SH/boot"="0/93"]:0.000003)[&"SH/boot"="88.6/65"]:0.002748)[&"SH/boot"="0/6"]:0.000002,"NAS_H_F02":0.002762)[&"SH/boot"="0/1"]:0.000002,("NAS_G_F05":0.002748,("NAS_G_F03":0.000002,"NAS_H_M02":0.002748)[&"SH/boot"="85.7/69"]:0.002762)[&"SH/boot"="0/7"]:0.000002)[&"SH/boot"="0/2"]:0.000002)[&"SH/boot"="0/1"]:0.000002,"NAS_D_F02":0.000002)[&"SH/boot"="0/2"]:0.000002,("NAS_P_F04":0.002748,"NAS_G_M04":0.005516)[&"SH/boot"="0/15"]:0.000002)[&"SH/boot"="0/8"]:0.000002,("NAS_D_M01":0.002748,((("NAS_H_F01":0,"NAS_Z_M04":0):0,"NAS_K_M05":0):0.000002,"NAS_K_M04":0.000002)[&"SH/boot"="0/70"]:0.000002)[&"SH/boot"="85.5/40"]:0.002748)[&"SH/boot"="78.6/97"]:0.002829,"NAS_H_M04":0.002702)[&"SH/boot"="96.6/100"]:0.014421,"NAS_P_F01":0.021168)[&"SH/boot"="83/97"]:0.00775,((((((((((((((((((((((((((((((((((((((("LAB_D_F01":0,"LAB_G_M01":0):0,"LAB_Z_F01":0):0,"LAB_Z_M02":0):0,"LAB_Z_F02":0):0,"LAB_P_F05":0):0,"LAB_K_F05":0):0,"LAB_P_F02":0):0,"LAB_H_F03":0):0,"LAB_Z_F04":0):0,"LAB_Z_F03":0):0,"LAB_P_M05":0):0,"LAB_Z_F06":0):0,"LAB_K_M02":0):0,"LAB_H_F02":0):0,"LAB_D_F04":0):0,"LAB_G_M05":0):0,"LAB_K_M05":0):0,"LAB_D_M01":0):0,"LAB_H_F04":0):0,"LAB_G_F01":0):0,"LAB_K_F04":0):0,"LAB_P_M04":0):0,"LAB_G_F04":0):0,"LAB_D_F05":0):0,"LAB_Z_M01":0):0,"LAB_G_F05":0):0,"LAB_K_F01":0):0,"LAB_Z_F

05":0):0,"LAB_D_M05":0):0,"LAB_P_F04":0):0,"LAB_H_F05":0):0.000002,"LAB_D_F03":0.000002)[&"SH/boot"="0/51"]:0.000002,("LAB_P_F03":0.002773,"LAB_Z_M03":0.002773)[&"SH/boot"="0/11"]:0.000002)[&"SH/boot"="0/8"]:0.000002,(("LAB_H_M02":0.000002,"LAB_P_F01":0.000002)[&"SH/boot"="0/74"]:0.000002,"LAB_P_M03":0.002782)[&"SH/boot"="85.7/99"]:0.002773)[&"SH/boot"="0/21"]:0.000002,("LAB_K_M01":0.000002,("LAB_P_M02":0.005567,"LAB_P_M01":0.005592)[&"SH/boot"="0/45"]:0.000002)[&"SH/boot"="85.5/74"]:0.002773)[&"SH/boot"="86/73"]:0.002773,("LAB_D_F02":0.000002,"LAB_D_M04":0.000002)[&"SH/boot"="0/72"]:0.000002)[&"SH/boot"="83.1/73"]:0.002773,("LAB_D_M02":0.000002,"LAB_H_M04":0.000002)[&"SH/boot"="0/79"]:0.000002)[&"SH/boot"="95.5/93"]:0.008381,((((((((((((("LAB_D_M03":0,"LAB_H_F01":0):0,"LAB_K_M03":0):0,"LAB_G_M04":0):0,"LAB_K_F02":0):0,"LAB_G_M03":0):0,"LAB_K_M04":0):0,"LAB_H_M01":0):0,"LAB_Z_M04":0):0,"LAB_H_M05":0):0.000002,"LAB_G_F03":0.000002)[&"SH/boot"="0/55"]:0.000002,("LAB_G_F02":0.002773,"LAB_H_M03":0.002773)[&"SH/boot"="0/11"]:0.000002)[&"SH/boot"="0/8"]:0.000002,"LAB_G_M02":0.002773)[&"SH/boot"="92.5/93"]:0.005592,"LAB_K_F03":0.000002)[&"SH/boot"="0/48"]:0.000002)[&"SH/boot"="98.5/100"]:0.022611,(("CAL_P_F08":0.008496,"CAL_G_F02":0.005566)[&"SH/boot"="89.4/99"]:0.008293,(((("CAL_K_F01":0,"CAL_P_F03":0):0.000002,"CAL_P_F02":0.000002)[&"SH/boot"="84.2/99"]:0.002748,(("CAL_K_F04":0.000002,"CAL_P_F09":0.000002)[&"SH/boot"="0/55"]:0.000002,(("CAL_K_F02":0.002748,"CAL_K_M01":0.002748)[&"SH/boot"="0/76"]:0.000002,"CAL_P_M01":0.000002)[&"SH/boot"="92.8/99"]:0.002748)[&"SH/boot"="0/60"]:0.000002)[&"SH/boot"="96/100"]:0.011333,((((((("CAL_D_M01":0,"CAL_H_M02":0):0,"CAL_H_F01":0):0,"CAL_Z_F02":0):0,"CAL_H_F05":0):0,"CAL_G_M04":0):0.000002,"CAL_G_M03":0.000002)[&"SH/boot"="92.8/100"]:0.002748,(("CAL_D_M02":0,"CAL_H_F04":0):0.000002,"CAL_G_F11":0.000002)[&"SH/boot"="0/65"]:0.000002)[&"SH/boot"="0/72"]:0.000002,(((("CAL_D_F07":0,"CAL_K_F08":0):0.000002,"CAL_G_F01":0.000003)[&"SH/boot"="81.5/100"]:0.002753,("CAL_G_M02":0.000002,"CAL_H_M05":0.000002)[&"SH/boot"="80.4/100"]:0.002749)[&"SH/boot"="77.9/98"]:0.002811,("CAL_G_F04":0.005516,("CAL_D_F05":0.002763,(("CAL_H_F02":0.002748,"CAL_Z_M01":0.002748)[&"SH/boot"="0/5"]:0.000002,(((("CAL_H_M03":0.000002,"CAL_G_F03":0.002758)[&"SH/boot"="85/99"]:0.002749,"CAL_D_F06":0.005546)[&"SH/boot"="0/13"]:0.000002,"CAL_D_F02":0.00276)[&"SH/boot"="0/1"]:0.000002,("CAL_D_F03":0.000002,("CAL_H_M04":0,("CAL_D_F04":0,("CAL_D_M03":0,("CAL_H_F03":0,("CAL_H_M01":0,("CAL_G_F13":0,("CAL_Z_F01":0,("CAL_G_M01":0,("CAL_G_F08":0,("CAL_K_F05":0,("CAL_D_F01":0,"CAL_G_F06":0):0):0):0):0):0):0):0):0):0):0.000002):0.000002)[&"SH/boot"="0/24"]:0.000002)[&"SH/boot"="0/2"]:0.000002)[&"SH/boot"="0/7"]:0.000002)[&"SH/boot"="0/30"]:0.002757)[&"SH/boot"="77.7/98"]:0.002745)[&"SH/boot"="83/94"]:0.00561)[&"SH/boot"="78.6/88"]:0.000002)[&"SH/boot"="0/88"]:0.011029)[&"SH/boot"="77.4/89"]:0.009546)[&"SH/boot"="79.7/83"]:0.007917)[&"SH/boot"="80.3/91"]:0.007123):0.0;

end;

Data S2. Cytochrome oxidase I phylogenetic tree in Nexus format.

#NEXUS

begin trees;

tree tree_1 = [&R]
(((("MIN_K_F05":0.00427,("MIN_G_M05":0.001711,("MIN_G_F03":0.001567,("MIN_Z_F02":0.00148
5,"MIN_G_F04":0.004729)[&"SH/boot"="0/88"]:0.000002)[&"SH/boot"="75.3/95"]:0.001436)[&"SH/
boot"="97.3/99"]:0.016859)[&"SH/boot"="86.9/99"]:0.031641,("MIN_K_F03":0.034754,("MIN_K_F0
1":0.010142,("MIN_K_M04":0.021144,("MIN_K_M02":0.010971,("MIN_G_F05":0.010732,("MIN_K_
M05":0.007906,("MIN_K_M03":0.000002,"MIN_G_M01":0.00304)[&"SH/boot"="60.3/100"]:0.00379
5)[&"SH/boot"="96.1/100"]:0.011928)[&"SH/boot"="79/74"]:0.003766)[&"SH/boot"="78.8/73"]:0.00
3563)[&"SH/boot"="0/72"]:0.000003)[&"SH/boot"="82/87"]:0.008527)[&"SH/boot"="0/64"]:0.00000
2)[&"SH/boot"="96.5/99"]:0.301323,("MIN_P_M05":0.000003,("MIN_P_M01":0.003645,(("MIN_G_
M02":0.001771,"MIN_H_F01":0.005613)[&"SH/boot"="76.6/99"]:0.00188,("MIN_D_F04":0.005116,(
"MIN_D_M02":0.000002,(("MIN_G_M03":0.001766,"MIN_D_F02":0.009368)[&"SH/boot"="78.2/49"
]:0.001722,((("MIN_D_M05":0.000002,"MIN_H_F02":0.000002)[&"SH/boot"="77.1/99"]:0.001886,("
MIN_D_M03":0.005004,("MIN_G_M04":0.001751,"MIN_Z_F03":0.003341)[&"SH/boot"="0/74"]:0.00
0002)[&"SH/boot"="71.9/100"]:0.001413)[&"SH/boot"="90.8/99"]:0.005475,(("MIN_P_F04":0.00525
7,("MIN_D_F01":0.003267,"MIN_D_M01":0.004725)[&"SH/boot"="68.5/93"]:0.00135)[&"SH/boot"=
"76.6/93"]:0.00206,("MIN_D_F03":0.007256,(("MIN_K_M01":0.008807,("MIN_P_F02":0.003057,("MI
N_H_F03":0.009762,("MIN_H_M02":0.001744,"MIN_Z_M02":0.001848)[&"SH/boot"="0/92"]:0.0000
02)[&"SH/boot"="87.6/100"]:0.004256)[&"SH/boot"="56.6/94"]:0.003621)[&"SH/boot"="76.8/96"]:0
.002427,((("MIN_D_F05":0.000002,"MIN_D_M04":0.000002)[&"SH/boot"="94.5/100"]:0.005216,("M
IN_K_F04":0.01058,"MIN_P_M04":0.003466)[&"SH/boot"="76.2/100"]:0.00166)[&"SH/boot"="72/91
"]:0.003508,("MIN_H_F05":0.008858,("MIN_P_F03":0.003519,(("MIN_Z_M01":0.00339,"MIN_P_M0
2":0.003505)[&"SH/boot"="0/37"]:0.000002,(("MIN_H_F04":0.000002,"MIN_P_F05":0.000002)[&"S
H/boot"="0/88"]:0.000002,(("MIN_H_M01":0.003493,"MIN_G_F01":0.001703)[&"SH/boot"="0/40"]:
0.000002,("MIN_K_F02":0.003474,("MIN_P_M03":0.003616,("MIN_P_F01":0.000002,"MIN_Z_F01":0
.001839)[&"SH/boot"="0/93"]:0.000002)[&"SH/boot"="83.9/98"]:0.001781)[&"SH/boot"="0/10"]:0.0
00002)[&"SH/boot"="0/5"]:0.000002)[&"SH/boot"="0/7"]:0.000002)[&"SH/boot"="0/10"]:0.000003)
[&"SH/boot"="81/80"]:0.001748)[&"SH/boot"="0/76"]:0.000003)[&"SH/boot"="74.4/92"]:0.001742)
[&"SH/boot"="0/75"]:0.000002)[&"SH/boot"="0/84"]:0.000002)[&"SH/boot"="96.3/99"]:0.00355)[&
"SH/boot"="0/83"]:0.000002)[&"SH/boot"="0/4"]:0.000002)[&"SH/boot"="0/9"]:0.000003)[&"SH/bo
ot"="85.8/53"]:0.001776)[&"SH/boot"="0/37"]:0.000002)[&"SH/boot"="79/41"]:0.001851)[&"SH/bo
ot"="93.1/94"]:0.244823)[&"SH/boot"="98.1/100"]:0.174311,(("MIN_G_F02":0.412483,(("LON_H_M
05":0.000002,("LON_G_M04":0,"LON_K_F03":0):0.000002)[&"SH/boot"="0/87"]:0.000002,(("LON_K
_M03":0.00872,("LON_Z_F05":0.014755,"LON_K_F02":0.018178)[&"SH/boot"="0/83"]:0.000593)[&"
SH/boot"="94/84"]:0.018084,("LON_H_M02":0.001881,(("LON_H_F01":0.001881,("LON_G_F02":0.0
00002,"LON_Z_F01":0.003409)[&"SH/boot"="85.6/98"]:0.001803)[&"SH/boot"="77.3/98"]:0.00271,(
"LON_G_F03":0.013483,((("LON_Z_M02":0.001573,"LON_G_M03":0.012238)[&"SH/boot"="76.6/66"
]:0.001513,(("LON_P_F02":0.003146,("LON_K_F05":0.00327,("LON_P_M05":0.009872,("LON_G_M01
":0.003357,("LON_G_M02":0.012733,"LON_Z_M03":0.002927)[&"SH/boot"="0/60"]:0.000002)[&"SH
/boot"="0/42"]:0.000002)[&"SH/boot"="86/69"]:0.001502)[&"SH/boot"="82.3/69"]:0.001518)[&"SH
/boot"="0/42"]:0.000003,(("LON_H_M04":0.00517,"LON_D_M03":0.008789)[&"SH/boot"="77.3/95"
]:0.001728,(("LON_D_M01":0.000002,("LON_G_F05":0.006878,"LON_G_F04":0.003387)[&"SH/boot"
="0/83"]:0.000002)[&"SH/boot"="92.7/92"]:0.001658,(("LON_Z_M05":0.000002,("LON_P_M01":0.00
0002,"LON_K_F04":0.001715)[&"SH/boot"="96.6/100"]:0.005493)[&"SH/boot"="83.1/89"]:0.001691
,(("LON_K_M01":0.002962,"LON_Z_F02":0.01337)[&"SH/boot"="75/58"]:0.001853,("LON_Z_F03":0.0

03388,("LON_K_F01":0.000002,"LON_D_M02":0.00166)[&"SH/boot"="0/72"]:0.000002)[&"SH/boot"
="91.9/96"]:0.003333)[&"SH/boot"="0/19"]:0.000002)[&"SH/boot"="0/22"]:0.000003)[&"SH/boot"=
"0/29"]:0.000002)[&"SH/boot"="84.4/84"]:0.001601)[&"SH/boot"="0/32"]:0.000003)[&"SH/boot"="
85.5/67"]:0.001566,("LON_Z_M04":0.009354,("LON_K_M02":0.008429,(("LON_H_F02":0.010828,("L
ON_K_M05":0.012557,"LON_P_M03":0.02987)[&"SH/boot"="0/94"]:0.000452)[&"SH/boot"="87.5/7
9"]:0.004784,(("LON_H_F04":0.001547,("LON_P_F04":0.002197,"LON_P_F03":0.004751)[&"SH/boot
"="33.9/97"]:0.000611)[&"SH/boot"="83.8/100"]:0.001649,("LON_H_M03":0.009705,("LON_P_F01":
0.002854,("LON_D_F01":0.001826,("LON_G_F01":0.001682,(("LON_Z_F04":0.001674,("LON_P_M02"
:0.000002,"LON_P_F05":0.000002)[&"SH/boot"="83.3/100"]:0.00166)[&"SH/boot"="0/9"]:0.000002,
(("LON_P_M04":0.001662,("LON_H_M01":0.001627,"LON_Z_M01":0.005129)[&"SH/boot"="0/27"]:0
.000002)[&"SH/boot"="0/6"]:0.000003,("LON_H_F03":0.000002,("LON_H_F05":0.001763,("LON_K_
M04":0.001714,"LON_G_M05":0.001794)[&"SH/boot"="0/77"]:0.000002)[&"SH/boot"="0/75"]:0.00
0002)[&"SH/boot"="0/67"]:0.000002)[&"SH/boot"="0/3"]:0.000002)[&"SH/boot"="0/23"]:0.000003)
[&"SH/boot"="75.8/57"]:0.001521)[&"SH/boot"="83.5/88"]:0.00325)[&"SH/boot"="91.3/100"]:0.003
421)[&"SH/boot"="0/57"]:0.000002)[&"SH/boot"="88.6/100"]:0.005005)[&"SH/boot"="48.1/52"]:0.0
04168)[&"SH/boot"="0/39"]:0.000002)[&"SH/boot"="0/45"]:0.000002)[&"SH/boot"="78.2/56"]:0.00
1605)[&"SH/boot"="0/71"]:0.00062)[&"SH/boot"="98.5/79"]:0.01626)[&"SH/boot"="96.5/99"]:0.022
194)[&"SH/boot"="94.3/99"]:0.020811)[&"SH/boot"="92.5/100"]:0.242266)[&"SH/boot"="64.7/94"]:
0.163572,(("LAB_H_F03":0.000002,(("LAB_D_M03":0.000002,("LAB_K_M05":0.000002,((("LAB_G_F0
2":0.001428,"LAB_Z_F06":0.001681)[&"SH/boot"="80.8/93"]:0.001646,("LAB_H_M03":0.00177,"LAB
_P_M03":0.011927)[&"SH/boot"="72.1/94"]:0.001441)[&"SH/boot"="0/58"]:0.000002,("LAB_K_F03"
:0.003161,(("LAB_P_F01":0.001513,"LAB_D_F01":0.00489)[&"SH/boot"="0/11"]:0.000003,(("LAB_G_
M03":0.000002,("LAB_H_M04":0,"LAB_K_M04":0):0.000002)[&"SH/boot"="0/86"]:0.000002,("LAB_
G_F05":0.005208,("LAB_D_M01":0.000002,("LAB_H_M05":0,("LAB_D_F02":0,"LAB_D_M02":0):0):0.0
00002)[&"SH/boot"="91.3/100"]:0.003111)[&"SH/boot"="0/7"]:0.000002)[&"SH/boot"="0/7"]:0.000
002)[&"SH/boot"="0/7"]:0.000002)[&"SH/boot"="85.7/78"]:0.001554)[&"SH/boot"="83.8/81"]:0.001
56)[&"SH/boot"="93.9/92"]:0.010592)[&"SH/boot"="98.8/99"]:0.037136,("LAB_K_M02":0.003021,("
LAB_Z_M02":0.003445,("LAB_G_M02":0.001777,(("LAB_P_F05":0.002768,("LAB_K_F01":0.005304,((
"LAB_K_M03":0.001689,"LAB_G_F04":0.00174)[&"SH/boot"="0/13"]:0.000002,(("LAB_G_M04":0.00
174,("LAB_H_F05":0.000002,("LAB_H_F01":0,"LAB_Z_F05":0):0.000002)[&"SH/boot"="84.6/100"]:0.
001696)[&"SH/boot"="0/12"]:0.000002,("LAB_D_F04":0.00174,("LAB_P_M02":0.00169,("LAB_K_M0
1":0.000002,"LAB_K_F05":0.000002)[&"SH/boot"="96.1/100"]:0.005364)[&"SH/boot"="0/29"]:0.000
002)[&"SH/boot"="0/5"]:0.000002)[&"SH/boot"="0/4"]:0.000002)[&"SH/boot"="0/64"]:0.000002)[&
"SH/boot"="75.2/81"]:0.002394)[&"SH/boot"="97.2/75"]:0.009928,("LAB_G_M01":0.005238,("LAB_
P_F03":0.001741,("LAB_K_F02":0.001645,(("LAB_D_F05":0.000002,("LAB_D_M04":0,("LAB_P_M04":
0,("LAB_K_F04":0,"LAB_P_M05":0):0):0):0.000002)[&"SH/boot"="0/86"]:0.000002,((("LAB_Z_F01":0.
001627,"LAB_Z_M03":0.000002)[&"SH/boot"="83.9/100"]:0.001653,(("LAB_Z_F03":0.00165,"LAB_H
_M01":0.001651)[&"SH/boot"="0/17"]:0.000002,("LAB_H_F02":0.001651,"LAB_D_M05":0.006559)[
&"SH/boot"="0/9"]:0.000002)[&"SH/boot"="0/14"]:0.000002)[&"SH/boot"="85.7/97"]:0.001627,(("L
AB_P_F02":0.003488,("LAB_Z_M04":0.000002,"LAB_H_F04":0.000002)[&"SH/boot"="79.5/100"]:0.0
01721)[&"SH/boot"="79.4/92"]:0.001577,("LAB_P_M01":0.003247,("LAB_H_M02":0.00148,(("LAB_D
_F03":0.003155,"LAB_G_M05":0.000002)[&"SH/boot"="84.2/73"]:0.001518,(("LAB_G_F03":0.00163
5,("LAB_Z_F04":0.000002,"LAB_Z_M01":0.000002)[&"SH/boot"="0/90"]:0.000002)[&"SH/boot"="86
/95"]:0.001542,("LAB_G_F01":0.001479,("LAB_P_F04":0.003049,"LAB_Z_F02":0.001456)[&"SH/boot
"="89.9/98"]:0.003048)[&"SH/boot"="0/45"]:0.000003)[&"SH/boot"="0/18"]:0.000002)[&"SH/boot"
="0/20"]:0.000002)[&"SH/boot"="81.7/60"]:0.001559)[&"SH/boot"="0/30"]:0.000003)[&"SH/boot"=
"0/18"]:0.000002)[&"SH/boot"="0/16"]:0.000002)[&"SH/boot"="0/19"]:0.000002)[&"SH/boot"="73.
3/87"]:0.001602)[&"SH/boot"="75/93"]:0.001859)[&"SH/boot"="76.2/94"]:0.001809)[&"SH/boot"="
0/32"]:0.000003)[&"SH/boot"="0/30"]:0.000002)[&"SH/boot"="0/26"]:0.000652)[&"SH/boot"="86.9

/85"]:0.014756)[&"SH/boot"="91.9/96"]:0.040117)[&"SH/boot"="99.4/100"]:0.341116,(("NAS_P_F0
1":0.132618,("NAS_D_F02":0.029916,(("NAS_G_M04":0.000002,"NAS_G_F04":0.000002)[&"SH/boot
"="92.9/100"]:0.011333,("NAS_P_F04":0.020708,(("NAS_P_F03":0.031809,"NAS_H_F02":0.018896)[
&"SH/boot"="96.4/98"]:0.017776,(("NAS_P_M02":0.004972,("NAS_H_M05":0.00112,"NAS_H_M04":
0.004346)[&"SH/boot"="92.4/100"]:0.007221)[&"SH/boot"="92.4/100"]:0.008255,((("NAS_D_F05":0.
023925,("NAS_P_M05":0.009205,("NAS_P_M04":0.002913,(("NAS_D_F01":0.004319,("NAS_G_M03"
:0.000002,"NAS_G_F03":0.000002)[&"SH/boot"="98.6/100"]:0.006638)[&"SH/boot"="0/41"]:0.0000
02,("NAS_P_M03":0.001354,("NAS_D_F04":0.005696,("NAS_G_F02":0.000002,"NAS_G_M02":0.0000
02)[&"SH/boot"="91.9/100"]:0.001356)[&"SH/boot"="0/31"]:0.000002)[&"SH/boot"="0/27"]:0.0000
03)[&"SH/boot"="76.6/95"]:0.001266)[&"SH/boot"="99/100"]:0.010135)[&"SH/boot"="66.1/94"]:0.0
0172)[&"SH/boot"="0/70"]:0.000002,(("NAS_Z_M05":0.002567,("NAS_G_M05":0.000002,"NAS_G_F
05":0.000002)[&"SH/boot"="84.1/100"]:0.001248)[&"SH/boot"="0/86"]:0.000002,((("NAS_D_M01":
0.000002,"NAS_G_M01":0.002503)[&"SH/boot"="99.8/100"]:0.009624,("NAS_H_F04":0.00142,"NAS
_Z_F03":0.004051)[&"SH/boot"="0/95"]:0.000002)[&"SH/boot"="79.3/55"]:0.001294,(("NAS_D_M04
":0.001317,"NAS_Z_F02":0.00435)[&"SH/boot"="0/97"]:0.000002,("NAS_Z_M03":0.005247,("NAS_D
_M03":0.000002,"NAS_P_M01":0.006092)[&"SH/boot"="82.8/82"]:0.001247)[&"SH/boot"="0/78"]:0
.000002)[&"SH/boot"="77.8/83"]:0.001305)[&"SH/boot"="77.3/54"]:0.001245)[&"SH/boot"="99.1/1
00"]:0.010002)[&"SH/boot"="74.1/48"]:0.002707,("NAS_K_F02":0.005248,("NAS_K_F03":0.003738,(
"NAS_Z_F05":0.001232,("NAS_Z_M01":0.005154,("NAS_H_F01":0.002603,(("NAS_P_F05":0.001284,(
("NAS_D_M02":0.003056,"NAS_Z_M04":0.004339)[&"SH/boot"="60.3/51"]:0.000727,("NAS_K_M05"
:0.001428,("NAS_Z_F01":0.003095,"NAS_K_M02":0.006103)[&"SH/boot"="50/99"]:0.000617)[&"SH/
boot"="55/99"]:0.001279)[&"SH/boot"="76.8/90"]:0.001432)[&"SH/boot"="77.2/68"]:0.001243,(("N
AS_Z_M02":0.001249,"NAS_H_M03":0.002845)[&"SH/boot"="0/6"]:0.000002,("NAS_K_F01":0.0000
02,("NAS_H_M01":0.000002,((("NAS_K_F04":0.000002,"NAS_H_F05":0.002565)[&"SH/boot"="83.6/
94"]:0.00125,(("NAS_K_F05":0.001466,"NAS_D_M05":0.008977)[&"SH/boot"="69.8/49"]:0.0011,("N
AS_H_F03":0.002454,"NAS_P_F02":0.003875)[&"SH/boot"="76.4/69"]:0.00125)[&"SH/boot"="0/10"]
:0.000002)[&"SH/boot"="0/8"]:0.000002,("NAS_D_F03":0.004065,("NAS_K_M01":0.001721,("NAS_Z
_F04":0.001249,("NAS_K_M03":0.003738,("NAS_K_M04":0.000345,"NAS_G_F01":0.01734)[&"SH/bo
ot"="76/66"]:0.002125)[&"SH/boot"="0/54"]:0.000002)[&"SH/boot"="0/56"]:0.000002)[&"SH/boot"
="84.8/65"]:0.001639)[&"SH/boot"="0/24"]:0.000002)[&"SH/boot"="0/5"]:0.000002)[&"SH/boot"="
0/4"]:0.000002)[&"SH/boot"="0/6"]:0.000002)[&"SH/boot"="0/7"]:0.000002)[&"SH/boot"="0/14"]:0
.000002)[&"SH/boot"="0/31"]:0.000002)[&"SH/boot"="84.5/47"]:0.001229)[&"SH/boot"="92.4/53"]:
0.001639)[&"SH/boot"="76.1/47"]:0.001156)[&"SH/boot"="93.6/98"]:0.005406)[&"SH/boot"="65.2/
69"]:0.001075)[&"SH/boot"="80.8/71"]:0.002985)[&"SH/boot"="45.1/93"]:0.00167)[&"SH/boot"="5
8.4/96"]:0.001041)[&"SH/boot"="94.8/100"]:0.055319)[&"SH/boot"="93.6/97"]:0.081858)[&"SH/bo
ot"="86.1/91"]:0.111296,(("CAL_P_F03":0.009947,"CAL_P_F09":0.000002)[&"SH/boot"="94.2/100"]:
0.119991,(((("CAL_P_F07":0.023226,("CAL_P_F06":0.03467,("CAL_G_M05":0.005238,("CAL_G_M06":
0.006258,"CAL_G_M07":0.00176)[&"SH/boot"="35.3/96"]:0.004411)[&"SH/boot"="97.3/100"]:0.027
893)[&"SH/boot"="73.6/99"]:0.014847)[&"SH/boot"="74.2/84"]:0.010767,(("CAL_G_F09":0.009829,"
CAL_K_F06":0.017331)[&"SH/boot"="77.4/100"]:0.007845,("CAL_P_F05":0.019535,("CAL_G_F10":0.
010468,("CAL_G_F12":0.00433,("CAL_P_F01":0.004879,("CAL_P_F04":0.00112,("CAL_G_F05":0.0046
55,"CAL_K_M02":0.003164)[&"SH/boot"="76.7/100"]:0.002624)[&"SH/boot"="72.4/100"]:0.001548)
[&"SH/boot"="70.4/100"]:0.002388)[&"SH/boot"="97.2/100"]:0.014095)[&"SH/boot"="89.1/100"]:0.
009826)[&"SH/boot"="92/100"]:0.020437)[&"SH/boot"="97.2/100"]:0.033542)[&"SH/boot"="60.8/8
4"]:0.017083,("CAL_D_M02":0.002416,("CAL_D_M03":0.004817,(("CAL_G_F08":0.003167,"CAL_P_M
01":0.003106)[&"SH/boot"="0/12"]:0.000002,("CAL_D_F03":0.001556,("CAL_G_F11":0.003063,(("CA
L_D_M01":0.003756,("CAL_K_F05":0.004413,("CAL_G_F01":0.004396,("CAL_H_F01":0.000002,"CAL_
D_F01":0.000002)[&"SH/boot"="79.9/100"]:0.001471)[&"SH/boot"="75.5/100"]:0.001356)[&"SH/bo
ot"="88.8/100"]:0.002957)[&"SH/boot"="88.8/100"]:0.002985,("CAL_D_F06":0.000002,("CAL_G_M0

1":0.003122,("CAL_G_M02":0.003253,((("CAL_H_F02":0.006003,"CAL_K_F08":0.006971)[&"SH/boot"
="48.8/97"]:0.000643,("CAL_Z_F01":0.001554,"CAL_H_M04":0.001574)[&"SH/boot"="78.5/100"]:0.0
01574)[&"SH/boot"="63.6/99"]:0.003967,("CAL_G_F13":0.004768,("CAL_H_M02":0.002718,("CAL_G
_F03":0.004158,(("CAL_Z_M01":0.001083,"CAL_D_F05":0.004202)[&"SH/boot"="74.5/86"]:0.002608
,(("CAL_H_F05":0.002535,("CAL_G_M04":0.002561,"CAL_Z_F02":0.006435)[&"SH/boot"="87.5/99"]:
0.002707)[&"SH/boot"="74.6/71"]:0.001331,(("CAL_G_M03":0.009586,("CAL_H_M01":0.004207,"CA
L_H_M05":0.001384)[&"SH/boot"="0/84"]:0.000002)[&"SH/boot"="81/81"]:0.001265,(("CAL_G_F06
":0.005209,"CAL_H_M03":0.002462)[&"SH/boot"="73.8/98"]:0.001381,("CAL_D_F07":0.000002,("CA
L_G_F04":0.004894,("CAL_D_F04":0.000002,("CAL_D_F02":0,("CAL_H_F04":0,"CAL_H_F03":0):0):0.0
00002)[&"SH/boot"="93.1/100"]:0.002503)[&"SH/boot"="0/90"]:0.000002)[&"SH/boot"="92.6/98"]:
0.002503)[&"SH/boot"="0/69"]:0.000003)[&"SH/boot"="0/75"]:0.000002)[&"SH/boot"="80.5/61"]:0.
00124)[&"SH/boot"="0/57"]:0.000003)[&"SH/boot"="73.5/64"]:0.001063)[&"SH/boot"="59.7/64"]:0.
000658)[&"SH/boot"="93.2/98"]:0.01221)[&"SH/boot"="100/100"]:0.078948)[&"SH/boot"="0/47"]:0
.000002)[&"SH/boot"="77.2/58"]:0.001439)[&"SH/boot"="88.6/59"]:0.003002)[&"SH/boot"="80.3/5
8"]:0.001509)[&"SH/boot"="0/8"]:0.000002)[&"SH/boot"="0/4"]:0.000002)[&"SH/boot"="0/5"]:0.00
0002)[&"SH/boot"="80/53"]:0.003945)[&"SH/boot"="99.5/100"]:0.071693)[&"SH/boot"="91.6/92"]:
0.095335,("COR_G_F02":0.001792,(("COR_H_F01":0.000003,(("COR_G_M02":0.001457,(("COR_H_M
04":0.001459,("COR_H_F03":0.00289,"COR_D_M01":0.002948)[&"SH/boot"="0/34"]:0.000002)[&"S
H/boot"="0/15"]:0.000002,("COR_P_F05":0.001458,("COR_P_M04":0.004449,("COR_Z_F04":0.00000
2,"COR_Z_F03":0.000002)[&"SH/boot"="88.7/100"]:0.002807)[&"SH/boot"="77.2/97"]:0.001465)[&"
SH/boot"="0/22"]:0.000002)[&"SH/boot"="0/16"]:0.000002)[&"SH/boot"="83.3/88"]:0.001496,((("C
OR_P_M01":0.003266,"COR_H_F05":0.008451)[&"SH/boot"="74.7/73"]:0.001495,("COR_Z_M03":0.
004722,("COR_Z_F01":0.009291,"COR_P_M02":0.0048)[&"SH/boot"="73.7/92"]:0.001559)[&"SH/bo
ot"="0/39"]:0.000003)[&"SH/boot"="0/18"]:0.000002,(("COR_K_F02":0.016252,("COR_G_F05":0.001
875,"COR_P_F04":0.00299)[&"SH/boot"="87.8/98"]:0.007885)[&"SH/boot"="2.3/69"]:0.001628,(("C
OR_H_F04":0.004707,("COR_D_M02":0.000002,"COR_Z_M02":0.000002)[&"SH/boot"="93.4/100"]:0
.008513)[&"SH/boot"="92.3/99"]:0.007486,("COR_K_F10":0.017362,("COR_D_F01":0.01742,"COR_P
_F03":0.006244)[&"SH/boot"="75.9/96"]:0.003467)[&"SH/boot"="0/89"]:0.000002)[&"SH/boot"="58
.5/92"]:0.000804)[&"SH/boot"="88.9/91"]:0.003897)[&"SH/boot"="0/29"]:0.000002)[&"SH/boot"="7
4.7/89"]:0.006545)[&"SH/boot"="99.8/100"]:0.038205,(("COR_H_M03":0.003379,("COR_D_M03":0.
001317,"COR_Z_M05":0.003773)[&"SH/boot"="59/99"]:0.000756)[&"SH/boot"="87.8/93"]:0.005895
,(("COR_D_F05":0.00421,("COR_D_F02":0.005432,((("COR_H_M05":0.00168,"COR_K_F09":0.006315)
[&"SH/boot"="69.1/97"]:0.001181,("COR_K_F05":0.004157,("COR_G_M03":0.001318,"COR_P_F01":
0.000002)[&"SH/boot"="82.1/99"]:0.001331)[&"SH/boot"="0/74"]:0.000002)[&"SH/boot"="85.3/89"
]:0.001367,("COR_D_F04":0.005434,("COR_P_M05":0.00545,("COR_H_M01":0.000002,("COR_K_F07
":0.002626,"COR_K_F06":0.002532)[&"SH/boot"="0/54"]:0.000002)[&"SH/boot"="0/54"]:0.000002)[
&"SH/boot"="32.3/98"]:0.001294)[&"SH/boot"="78.1/100"]:0.001335)[&"SH/boot"="0/78"]:0.00000
2)[&"SH/boot"="65/85"]:0.000932)[&"SH/boot"="98.4/100"]:0.014201,("COR_K_F08":0.005967,("CO
R_D_M05":0.002731,(("COR_Z_M04":0.001399,(("COR_P_F02":0.002755,"COR_G_M01":0.004213)[
&"SH/boot"="0/16"]:0.000002,("COR_Z_M01":0.002738,"COR_G_M05":0.002907)[&"SH/boot"="0/1
2"]:0.000002)[&"SH/boot"="0/10"]:0.000002)[&"SH/boot"="85.4/63"]:0.001365,("COR_K_F04":0.00
0002,((("COR_D_F03":0.000002,"COR_G_F03":0.001333)[&"SH/boot"="85/99"]:0.001333,("COR_K_F
03":0.002695,("COR_Z_F05":0.00559,"COR_G_M04":0.002613)[&"SH/boot"="0/78"]:0.000003)[&"S
H/boot"="83.6/81"]:0.001354)[&"SH/boot"="0/12"]:0.000002,(("COR_H_M02":0.007262,("COR_G_F
04":0.000002,"COR_G_F01":0.001251)[&"SH/boot"="84.3/96"]:0.001303)[&"SH/boot"="0/33"]:0.00
0002,("COR_K_F01":0.00133,("COR_Z_F02":0.000002,("COR_D_M04":0.000002,"COR_P_M03":0.000
002)[&"SH/boot"="92.2/100"]:0.001355)[&"SH/boot"="0/80"]:0.000002)[&"SH/boot"="83.7/98"]:0.0
01333)[&"SH/boot"="0/4"]:0.000002)[&"SH/boot"="0/6"]:0.000002)[&"SH/boot"="0/7"]:0.000003)[
&"SH/boot"="0/25"]:0.000002)[&"SH/boot"="80/58"]:0.002707)[&"SH/boot"="90.6/94"]:0.005875)[

```
&"SH/boot"="90.8/100"]:0.00424)[&"SH/boot"="87.8/94"]:0.010433)[&"SH/boot"="62.4/97"]:0.010
714)[&"SH/boot"="90.9/99"]:0.086559)[&"SH/boot"="94.1/99"]:0.132472)[&"SH/boot"="98.1/100"]:
0.262067)[&"SH/boot"="93.6/94"]:0.148787)[&"SH/boot"="92.3/97"]:0.019099)[&"SH/boot"="98.1/
100"]:0.174311):0.0;

end;
```

Data S3. Combined phylogenetic tree (internal transcribed spacer 2 and cytochrome oxidase I) in Nexus format.

#NEXUS

begin trees;

tree tree_1 = [&R]
(("MIN_G_F02":0.259969,(("MIN_Z_F03":0.001961,(("MIN_K_F05":0.002823,("MIN_G_M05":0.0011
07,("MIN_G_F03":0.00101,("MIN_G_F04":0.003025,"MIN_Z_F02":0.025064)[&"SH/boot"="0/56"]:0.
000003)[&"SH/boot"="75.1/83"]:0.000922)[&"SH/boot"="98/99"]:0.010707)[&"SH/boot"="83.7/99"]
:0.001035,("MIN_K_F03":0.022485,("MIN_K_F01":0.006541,("MIN_K_M04":0.013639,("MIN_K_M02
":0.007104,("MIN_G_F05":0.012623,("MIN_K_M05":0.006868,("MIN_G_M01":0.001946,"MIN_K_M
03":0.000002)[&"SH/boot"="73.3/100"]:0.002444)[&"SH/boot"="95.5/100"]:0.007665)[&"SH/boot"=
"78.6/87"]:0.002481)[&"SH/boot"="79.3/85"]:0.002283)[&"SH/boot"="0/68"]:0.000003)[&"SH/boot"
="75.7/86"]:0.005497)[&"SH/boot"="12.1/90"]:0.019196)[&"SH/boot"="100/100"]:0.400879)[&"SH/
boot"="88.2/95"]:0.015952,(("MIN_D_M03":0.003195,"MIN_G_M04":0.00297)[&"SH/boot"="0/46"]
:0.000002,("MIN_H_F02":0.001874,("MIN_D_M05":0.000003,(("MIN_P_M01":0.005256,("MIN_D_M
02":0.000002,("MIN_G_M03":0.004109,("MIN_D_F04":0.008905,(("MIN_D_F02":0.006143,"MIN_P_
M05":0.001206)[&"SH/boot"="92.4/100"]:0.0018,("MIN_G_M02":0.001126,"MIN_H_F01":0.003594)
[&"SH/boot"="78.1/100"]:0.001221)[&"SH/boot"="81.4/99"]:0.001118)[&"SH/boot"="92/96"]:0.003
663)[&"SH/boot"="0/10"]:0.000002)[&"SH/boot"="0/8"]:0.000002)[&"SH/boot"="0/44"]:0.000003,("
MIN_K_M01":0.007467,("MIN_H_F05":0.005692,(("MIN_P_M03":0.00244,"MIN_Z_F01":0.001383)[
&"SH/boot"="71.6/96"]:0.001004,("MIN_P_F03":0.002246,(("MIN_G_F01":0.001087,"MIN_P_F01":0
.001137)[&"SH/boot"="0/77"]:0.000002,("MIN_K_F02":0.006213,(("MIN_H_F04":0.000002,"MIN_P_
F05":0.000002)[&"SH/boot"="0/51"]:0.000002,(("MIN_H_M01":0.002228,"MIN_Z_M01":0.003958)[
&"SH/boot"="0/20"]:0.000002,("MIN_P_M02":0.002236,((("MIN_D_F05":0.00178,"MIN_D_M04":0.0
05788)[&"SH/boot"="91.8/99"]:0.002229,("MIN_K_F04":0.00679,"MIN_P_M04":0.003976)[&"SH/bo
ot"="89.1/100"]:0.002239)[&"SH/boot"="78.5/88"]:0.002238,(("MIN_D_F03":0.006403,("MIN_P_F04
":0.005138,("MIN_D_F01":0.002088,"MIN_D_M01":0.003022)[&"SH/boot"="70.4/69"]:0.000866)[&"
SH/boot"="82.7/69"]:0.001324)[&"SH/boot"="0/77"]:0.000002,("MIN_P_F02":0.004107,("MIN_H_F0
3":0.006261,("MIN_H_M02":0.001115,"MIN_Z_M02":0.004941)[&"SH/boot"="92.4/100"]:0.003772)
[&"SH/boot"="88.3/100"]:0.002353)[&"SH/boot"="92.9/98"]:0.003474)[&"SH/boot"="0/84"]:0.0000
02)[&"SH/boot"="87.2/62"]:0.002243)[&"SH/boot"="0/10"]:0.000003)[&"SH/boot"="0/32"]:0.00000
2)[&"SH/boot"="77.6/54"]:0.001774)[&"SH/boot"="79.1/94"]:0.001917)[&"SH/boot"="84.7/90"]:0.0
01875)[&"SH/boot"="0/62"]:0.000002)[&"SH/boot"="77/96"]:0.001115)[&"SH/boot"="73.9/97"]:0.0
01018)[&"SH/boot"="91.6/97"]:0.00242)[&"SH/boot"="95.8/96"]:0.004494)[&"SH/boot"="40.3/36"]:
0.001875)[&"SH/boot"="86/93"]:0.002102)[&"SH/boot"="86.5/96"]:0.006932)[&"SH/boot"="99.8/1
00"]:0.445901)[&"SH/boot"="66/86"]:0.047239,((("LON_G_M04":0.001938,"LON_H_M05":0.003157
)[&"SH/boot"="70.3/97"]:0.002089,("LON_K_F03":0.000002,(("LON_K_M03":0.011937,("LON_K_F02
":0.01277,"LON_Z_F05":0.010431)[&"SH/boot"="55.6/96"]:0.00047)[&"SH/boot"="92.6/84"]:0.0118
23,("LON_H_M02":0.001201,(("LON_K_M02":0.003774,"LON_P_F01":0.00691)[&"SH/boot"="22.4/4
7"]:0.001815,("LON_H_F02":0.01285,((("LON_G_F04":0.002349,"LON_G_F05":0.005692)[&"SH/boot
"="84.2/83"]:0.002106,("LON_G_M03":0.009878,"LON_Z_M02":0.001016)[&"SH/boot"="77.9/88"]:0
.000972)[&"SH/boot"="0/37"]:0.000003,("LON_G_M01":0.006265,("LON_P_F02":0.004046,(("LON_P
_M03":0.025262,(("LON_K_F05":0.004101,"LON_Z_M04":0.007151)[&"SH/boot"="0/66"]:0.000003,(
("LON_Z_F02":0.009953,("LON_K_F04":0.001113,"LON_P_M01":0.004097)[&"SH/boot"="93.8/99"]:
0.004678)[&"SH/boot"="0/82"]:0.000002,("LON_D_M01":0.001074,("LON_D_M03":0.006787,("LON
_H_M04":0.0045,("LON_P_M05":0.006855,"LON_Z_F03":0.003483)[&"SH/boot"="81/96"]:0.002148)
[&"SH/boot"="44.1/89"]:0.000981)[&"SH/boot"="84.3/90"]:0.000985)[&"SH/boot"="0/62"]:0.00000

3)[&"SH/boot"="85.2/81"]:0.00103)[&"SH/boot"="79.8/80"]:0.000996)[&"SH/boot"="80.4/87"]:0.00
1013,(("LON_G_F03":0.011004,("LON_D_M02":0.003061,"LON_K_F01":0.001993)[&"SH/boot"="87.1
/100"]:0.003179)[&"SH/boot"="0/70"]:0.000002,(("LON_Z_M03":0.003958,("LON_G_M02":0.008593
,("LON_H_F01":0.003183,("LON_G_F02":0.003046,"LON_Z_F01":0.003179)[&"SH/boot"="84.2/98"]:
0.001151)[&"SH/boot"="84.4/99"]:0.002262)[&"SH/boot"="1.5/65"]:0.002075)[&"SH/boot"="0/80"]:
0.000002,(("LON_K_M01":0.005186,"LON_Z_M05":0.001163)[&"SH/boot"="0/66"]:0.000002,(("LON
_P_F03":0.00305,"LON_P_F04":0.001599)[&"SH/boot"="0/100"]:0.00024,("LON_H_F04":0.001955,("
LON_H_M03":0.006276,("LON_K_M05":0.012413,("LON_D_F01":0.002024,("LON_H_M01":0.001052
,("LON_P_F05":0.001072,("LON_Z_F04":0.002073,("LON_G_M05":0.002149,("LON_H_F05":0.002104
,(("LON_G_F01":0.003077,("LON_H_F03":0.000983,"LON_K_M04":0.001086)[&"SH/boot"="94.1/99"
]:0.001985)[&"SH/boot"="0/35"]:0.000002,("LON_P_M02":0.001072,("LON_P_M04":0.001072,"LON
_Z_M01":0.003317)[&"SH/boot"="0/35"]:0.000002)[&"SH/boot"="0/39"]:0.000003)[&"SH/boot"="0/
25"]:0.000002)[&"SH/boot"="80.9/98"]:0.001017)[&"SH/boot"="77.1/98"]:0.000987)[&"SH/boot"="
79.3/99"]:0.001004)[&"SH/boot"="0/70"]:0.000003)[&"SH/boot"="82.5/99"]:0.001059)[&"SH/boot"
="95.8/99"]:0.004317)[&"SH/boot"="0/64"]:0.000003)[&"SH/boot"="80.6/84"]:0.000977)[&"SH/boo
t"="0/63"]:0.000002)[&"SH/boot"="98.6/98"]:0.005365)[&"SH/boot"="80.5/89"]:0.00099)[&"SH/boo
t"="74.7/86"]:0.000992)[&"SH/boot"="0/59"]:0.000003)[&"SH/boot"="79.2/77"]:0.001015)[&"SH/bo
ot"="84.8/98"]:0.001986)[&"SH/boot"="80/90"]:0.000986)[&"SH/boot"="79/86"]:0.001072)[&"SH/b
oot"="0/34"]:0.000002)[&"SH/boot"="98/97"]:0.012382)[&"SH/boot"="94.7/98"]:0.014427)[&"SH/b
oot"="97.4/87"]:0.013541)[&"SH/boot"="77.1/86"]:0.001135)[&"SH/boot"="98.5/99"]:0.170141,((("
LAB_P_M02":0.00107,("LAB_G_M04":0.001104,("LAB_H_F01":0.001075,("LAB_K_M03":0.001069,("L
AB_D_M03":0.001563,("LAB_G_F02":0.002914,("LAB_H_M03":0.003052,("LAB_H_M05":0.001973,((
"LAB_G_M03":0.000002,"LAB_K_M04":0.000002)[&"SH/boot"="0/25"]:0.000002,("LAB_K_F03":0.00
2004,(("LAB_H_M04":0.000003,("LAB_D_M02":0.000002,("LAB_D_F02":0.000002,"LAB_D_M01":0.0
01022)[&"SH/boot"="83.1/86"]:0.00101)[&"SH/boot"="93.8/93"]:0.001973)[&"SH/boot"="0/70"]:0.0
00002,(("LAB_D_F01":0.003104,"LAB_G_F05":0.003318)[&"SH/boot"="0/72"]:0.000003,(("LAB_K_M
05":0.001006,"LAB_Z_F06":0.002033)[&"SH/boot"="80.1/34"]:0.000976,("LAB_P_F01":0.000957,"LA
B_P_M03":0.010606)[&"SH/boot"="69.8/36"]:0.00103)[&"SH/boot"="0/47"]:0.000002)[&"SH/boot"
="90.9/85"]:0.002057)[&"SH/boot"="95.3/98"]:0.003081)[&"SH/boot"="91/98"]:0.002064)[&"SH/bo
ot"="0/78"]:0.000003)[&"SH/boot"="87.4/96"]:0.001009)[&"SH/boot"="0/85"]:0.000003)[&"SH/boo
t"="83.7/95"]:0.006338)[&"SH/boot"="100/100"]:0.031274)[&"SH/boot"="0/79"]:0.000003)[&"SH/b
oot"="0/48"]:0.000002)[&"SH/boot"="95.3/96"]:0.004269)[&"SH/boot"="25.8/93"]:0.00106,(("LAB_
D_F04":0.001104,(("LAB_H_F05":0.000002,"LAB_Z_F05":0.000002)[&"SH/boot"="84.1/83"]:0.00107
5,("LAB_K_F05":0.000002,"LAB_K_M01":0.001033)[&"SH/boot"="96.6/99"]:0.003401)[&"SH/boot"="
0/16"]:0.000002)[&"SH/boot"="0/7"]:0.000002,("LAB_G_F04":0.001104,("LAB_K_F01":0.003359,("LA
B_P_F05":0.00047,("LAB_H_M02":0.001965,(("LAB_D_F03":0.002001,"LAB_G_M05":0.000002)[&"SH
/boot"="88.1/94"]:0.000963,(("LAB_G_F01":0.000938,("LAB_P_F04":0.001931,"LAB_Z_F02":0.00092
9)[&"SH/boot"="88.7/99"]:0.00193)[&"SH/boot"="0/44"]:0.000003,((("LAB_H_F04":0.001119,"LAB_
P_F02":0.002249)[&"SH/boot"="74.9/94"]:0.000955,("LAB_Z_F04":0.000002,"LAB_Z_M01":0.000002
)[&"SH/boot"="0/99"]:0.000003)[&"SH/boot"="27.3/18"]:0.000977,(("LAB_P_F03":0.002148,("LAB_
G_M01":0.003348,("LAB_K_M02":0.002671,("LAB_H_F03":0.034508,"LAB_Z_M02":0.001163)[&"SH/
boot"="38/97"]:0.001093)[&"SH/boot"="70.3/97"]:0.00107)[&"SH/boot"="79.3/95"]:0.001203)[&"S
H/boot"="74.9/89"]:0.001011,(("LAB_D_M05":0.004186,("LAB_H_F02":0.001053,("LAB_Z_F03":0.00
1053,("LAB_Z_F01":0.001039,"LAB_Z_M03":0.001032)[&"SH/boot"="87.5/100"]:0.001055)[&"SH/bo
ot"="0/51"]:0.000002)[&"SH/boot"="0/60"]:0.000002)[&"SH/boot"="85.3/97"]:0.001035,(("LAB_K_F
04":0.000002,("LAB_P_M04":0,("LAB_D_F05":0,"LAB_P_M05":0):0):0.000002)[&"SH/boot"="0/79"]:
0.000002,("LAB_D_M04":0.001022,("LAB_P_M01":0.003114,("LAB_G_M02":0.005474,(("LAB_G_F03
":0.002223,"LAB_Z_M04":0.001147)[&"SH/boot"="79.3/91"]:0.000994,("LAB_H_M01":0.002096,"LA
B_K_F02":0.001042)[&"SH/boot"="0/47"]:0.000002)[&"SH/boot"="0/61"]:0.000003)[&"SH/boot"="9

6.4/99"]:0.005308)[&"SH/boot"="79.9/95"]:0.002157)[&"SH/boot"="0/87"]:0.000002)[&"SH/boot"="
0/77"]:0.000002)[&"SH/boot"="0/25"]:0.000003)[&"SH/boot"="85.1/33"]:0.00099)[&"SH/boot"="0/
13"]:0.000003)[&"SH/boot"="0/16"]:0.000003)[&"SH/boot"="0/59"]:0.000003)[&"SH/boot"="98.4/9
8"]:0.006936)[&"SH/boot"="79.8/93"]:0.00278)[&"SH/boot"="0/46"]:0.000003)[&"SH/boot"="0/12"]
:0.000003)[&"SH/boot"="83.4/52"]:0.002101)[&"SH/boot"="100/100"]:0.29431,(("NAS_P_F01":0.10
9762,("NAS_D_F02":0.011838,("NAS_D_F05":0.006947,(((("NAS_G_F04":0.000002,"NAS_G_M04":0.
001993)[&"SH/boot"="99.2/100"]:0.0092,(("NAS_P_F04":0.013625,("NAS_H_F02":0.013867,"NAS_P
_F03":0.019598)[&"SH/boot"="95.4/100"]:0.011322)[&"SH/boot"="42.8/71"]:0.001791,("NAS_H_M0
4":0.004937,("NAS_H_M05":0.000726,"NAS_P_M02":0.007642)[&"SH/boot"="48.1/95"]:0.001997)[
&"SH/boot"="94.1/100"]:0.007579)[&"SH/boot"="11.9/48"]:0.001936)[&"SH/boot"="58.2/54"]:0.00
0653,(("NAS_D_M01":0.002,"NAS_G_M01":0.001588)[&"SH/boot"="96.7/99"]:0.006289,(("NAS_Z_
M05":0.001637,("NAS_G_F05":0.000989,"NAS_G_M05":0.000002)[&"SH/boot"="85.5/100"]:0.00079
5)[&"SH/boot"="0/94"]:0.000002,(("NAS_H_F04":0.001017,"NAS_Z_F03":0.002666)[&"SH/boot"="70
.4/98"]:0.000738,("NAS_D_M04":0.00085,("NAS_Z_M03":0.003348,("NAS_D_M03":0.000002,("NAS_
P_M01":0.004853,"NAS_Z_F02":0.003581)[&"SH/boot"="75.9/87"]:0.000987)[&"SH/boot"="83.3/88
"]:0.000787)[&"SH/boot"="0/85"]:0.000002)[&"SH/boot"="80.6/98"]:0.000839)[&"SH/boot"="46.6/7
8"]:0.000793)[&"SH/boot"="36.8/79"]:0.001384)[&"SH/boot"="95.3/99"]:0.004172)[&"SH/boot"="7
5.2/53"]:0.000876,("NAS_P_M05":0.005909,(("NAS_P_M04":0.001863,(("NAS_D_F01":0.002756,("N
AS_G_F03":0.001006,"NAS_G_M03":0.000002)[&"SH/boot"="98.8/100"]:0.004239)[&"SH/boot"="0/
60"]:0.000002,("NAS_P_M03":0.000863,("NAS_D_F04":0.003636,("NAS_G_F02":0.003019,"NAS_G_
M02":0.000003)[&"SH/boot"="92.3/100"]:0.000865)[&"SH/boot"="0/44"]:0.000002)[&"SH/boot"="0
/46"]:0.000002)[&"SH/boot"="75.2/91"]:0.000798)[&"SH/boot"="98.8/100"]:0.006406,("NAS_G_F01
":0.010384,("NAS_K_M04":0.00257,(("NAS_K_M03":0.002392,"NAS_Z_F04":0.0008)[&"SH/boot"="0
/68"]:0.000002,("NAS_K_M01":0.001102,("NAS_H_M03":0.001824,((("NAS_H_F01":0.001671,("NAS_
D_M05":0.006449,"NAS_P_F05":0.001643)[&"SH/boot"="87/98"]:0.000994)[&"SH/boot"="85.8/91"]
:0.000995,("NAS_D_M02":0.00109,(("NAS_K_M02":0.00374,"NAS_Z_F01":0.002866)[&"SH/boot"="0
/98"]:0.000003,("NAS_K_M05":0.001451,"NAS_Z_M04":0.003581)[&"SH/boot"="58.6/98"]:0.000995
)[&"SH/boot"="80.8/98"]:0.001699)[&"SH/boot"="81/97"]:0.001406)[&"SH/boot"="0/3"]:0.000002,(
("NAS_K_F01":0.000002,(("NAS_H_F05":0.002629,"NAS_K_F04":0.000003)[&"SH/boot"="84.9/96"]:0
.000801,("NAS_K_F05":0.001639,("NAS_H_F03":0.001572,"NAS_P_F02":0.002483)[&"SH/boot"="79.
1/99"]:0.000803)[&"SH/boot"="0/45"]:0.000002)[&"SH/boot"="0/8"]:0.000002)[&"SH/boot"="0/2"]:
0.000002,("NAS_H_M01":0.000002,(("NAS_D_F03":0.002601,"NAS_Z_M02":0.0008)[&"SH/boot"="0
/14"]:0.000002,("NAS_Z_M01":0.003297,("NAS_Z_F05":0.000861,("NAS_K_F02":0.00409,"NAS_K_F0
3":0.002468)[&"SH/boot"="64.5/85"]:0.001051)[&"SH/boot"="75.2/79"]:0.00071)[&"SH/boot"="0/4
0"]:0.000003)[&"SH/boot"="0/12"]:0.000002)[&"SH/boot"="0/5"]:0.000002)[&"SH/boot"="0/5"]:0.0
00002)[&"SH/boot"="0/28"]:0.000002)[&"SH/boot"="85.2/90"]:0.001051)[&"SH/boot"="0/90"]:0.00
0002)[&"SH/boot"="0/81"]:0.000002)[&"SH/boot"="43.1/92"]:0.002661)[&"SH/boot"="83.6/99"]:0.0
03242)[&"SH/boot"="0/82"]:0.000002)[&"SH/boot"="59.4/94"]:0.001102)[&"SH/boot"="89.5/53"]:0.
008265)[&"SH/boot"="74.3/98"]:0.040106)[&"SH/boot"="98.8/100"]:0.060132)[&"SH/boot"="85.6/
97"]:0.073635,(("CAL_P_F03":0.00735,"CAL_P_F09":0.000002)[&"SH/boot"="92/100"]:0.076573,(("C
AL_P_M01":0.002972,(("CAL_D_M02":0.004034,"CAL_G_F11":0.001936)[&"SH/boot"="75.2/96"]:0.0
00996,(("CAL_D_M03":0.003067,"CAL_G_F08":0.002018)[&"SH/boot"="0/73"]:0.000003,("CAL_D_F0
3":0.000986,(("CAL_D_M01":0.005375,("CAL_K_F05":0.002799,("CAL_G_F01":0.005847,("CAL_D_F0
1":0.000003,"CAL_H_F01":0.002994)[&"SH/boot"="73.6/88"]:0.000931)[&"SH/boot"="77.7/88"]:0.0
00857)[&"SH/boot"="90.6/89"]:0.001873)[&"SH/boot"="88.9/99"]:0.001893,("CAL_D_F06":0.002021
,("CAL_G_M01":0.001974,("CAL_G_M02":0.005123,((("CAL_H_F02":0.004809,"CAL_K_F08":0.007487
)[&"SH/boot"="26.6/98"]:0.000406,("CAL_H_M04":0.001,"CAL_Z_F01":0.000986)[&"SH/boot"="77.5
/100"]:0.001021)[&"SH/boot"="63.4/99"]:0.002599,("CAL_H_M01":0.002492,("CAL_H_M05":0.0047
49,(("CAL_D_F05":0.004364,(("CAL_D_F07":0.003066,"CAL_G_F04":0.005069)[&"SH/boot"="0/79"]:0

.000002,(("CAL_D_F02":0.000986,"CAL_H_F04":0.001983)[&"SH/boot"="0/84"]:0.000002,("CAL_D_F
04":0.000002,"CAL_H_F03":0.000002)[&"SH/boot"="0/69"]:0.000003)[&"SH/boot"="91.8/98"]:0.001
576)[&"SH/boot"="89.6/99"]:0.001578)[&"SH/boot"="0/50"]:0.000003,("CAL_G_F06":0.004068,("CA
L_G_F13":0.004082,(("CAL_G_F03":0.003637,"CAL_H_M03":0.003147)[&"SH/boot"="87/54"]:0.0009
84,("CAL_Z_M01":0.002313,("CAL_H_M02":0.002371,("CAL_G_M03":0.00754,("CAL_H_F05":0.00225
2,("CAL_G_M04":0.001618,"CAL_Z_F02":0.004091)[&"SH/boot"="85.9/100"]:0.001664)[&"SH/boot"
="75.3/99"]:0.000943)[&"SH/boot"="0/95"]:0.000002)[&"SH/boot"="91.9/97"]:0.001978)[&"SH/boo
t"="86.4/97"]:0.000983)[&"SH/boot"="0/41"]:0.000003)[&"SH/boot"="81.2/38"]:0.000781)[&"SH/bo
ot"="0/25"]:0.000003)[&"SH/boot"="0/41"]:0.000003)[&"SH/boot"="88/80"]:0.000965)[&"SH/boot"
="89/100"]:0.008036)[&"SH/boot"="100/100"]:0.051339)[&"SH/boot"="0/77"]:0.000002)[&"SH/boo
t"="76.6/98"]:0.000912)[&"SH/boot"="87.1/98"]:0.0019)[&"SH/boot"="81/96"]:0.000956)[&"SH/boo
t"="0/59"]:0.000002)[&"SH/boot"="70.3/93"]:0.000996)[&"SH/boot"="97.9/95"]:0.005559)[&"SH/bo
ot"="98.9/93"]:0.029234,((("CAL_P_F06":0.026069,("CAL_G_M05":0.004922,("CAL_G_M06":0.00572
5,"CAL_G_M07":0.001137)[&"SH/boot"="54.1/99"]:0.002967)[&"SH/boot"="34.5/89"]:0.016137)[&"
SH/boot"="92/98"]:0.010749,("CAL_P_F07":0.016129,("CAL_G_F09":0.006206,("CAL_K_F06":0.0112
65,("CAL_P_F05":0.011474,("CAL_G_F10":0.008451,("CAL_G_F12":0.008338,("CAL_P_F01":0.008612
,("CAL_P_F04":0.000789,("CAL_G_F05":0.00292,"CAL_K_M02":0.001979)[&"SH/boot"="78/100"]:0.0
01573)[&"SH/boot"="75.4/100"]:0.000974)[&"SH/boot"="69.6/100"]:0.001348)[&"SH/boot"="95.1/
100"]:0.008898)[&"SH/boot"="91/98"]:0.006972)[&"SH/boot"="99.4/100"]:0.018043)[&"SH/boot"="
94.3/51"]:0.003584)[&"SH/boot"="99.6/99"]:0.031883)[&"SH/boot"="90.4/8"]:0.001765)[&"SH/boot
"="65.3/11"]:0.011485,("COR_G_F02":0.001145,(("COR_H_F01":0.001753,(("COR_H_F03":0.001851,(
("COR_D_M01":0.001889,"COR_G_M02":0.000935)[&"SH/boot"="0/14"]:0.000003,("COR_H_M04":0
.000936,("COR_P_F05":0.000936,("COR_P_M04":0.004617,("COR_Z_F03":0.000002,"COR_Z_F04":0.
000002)[&"SH/boot"="86.8/100"]:0.001804)[&"SH/boot"="77.1/98"]:0.000941)[&"SH/boot"="0/9"]:
0.000002)[&"SH/boot"="0/6"]:0.000002)[&"SH/boot"="0/14"]:0.000003)[&"SH/boot"="84.5/85"]:0.0
0096,((("COR_H_F05":0.005418,"COR_P_M01":0.003864)[&"SH/boot"="75.7/88"]:0.000961,("COR_Z
_M03":0.003036,("COR_P_M02":0.00309,"COR_Z_F01":0.005967)[&"SH/boot"="77.3/96"]:0.001008
)[&"SH/boot"="0/47"]:0.000002)[&"SH/boot"="0/20"]:0.000003,("COR_K_F02":0.011035,(("COR_G_
F05":0.001141,"COR_P_F04":0.001967)[&"SH/boot"="97/99"]:0.005569,(("COR_K_F10":0.011115,("
COR_D_F01":0.011065,"COR_P_F03":0.005786)[&"SH/boot"="75.7/98"]:0.002184)[&"SH/boot"="0/9
3"]:0.000002,("COR_H_F04":0.002905,("COR_D_M02":0.000002,"COR_Z_M02":0.000002)[&"SH/boo
t"="92.2/100"]:0.005559)[&"SH/boot"="92.6/98"]:0.004728)[&"SH/boot"="0/91"]:0.000002)[&"SH/b
oot"="0/47"]:0.000965)[&"SH/boot"="89.1/94"]:0.002061)[&"SH/boot"="0/30"]:0.000002)[&"SH/bo
ot"="86.6/93"]:0.004205)[&"SH/boot"="99.9/100"]:0.024678,(("COR_H_M03":0.002138,("COR_D_M
03":0.000842,"COR_Z_M05":0.002408)[&"SH/boot"="60/96"]:0.000507)[&"SH/boot"="89.7/89"]:0.0
03717,(("COR_D_F05":0.008172,("COR_D_F02":0.003499,(("COR_D_F04":0.003502,("COR_P_M05":0
.005278,("COR_H_M01":0.000002,("COR_K_F06":0.00163,"COR_K_F07":0.001691)[&"SH/boot"="0/5
2"]:0.000003)[&"SH/boot"="0/52"]:0.000002)[&"SH/boot"="41.9/97"]:0.000837)[&"SH/boot"="77.2/
99"]:0.000859,(("COR_H_M05":0.001095,"COR_K_F09":0.004072)[&"SH/boot"="70.4/99"]:0.000757,
("COR_K_F05":0.002688,("COR_G_M03":0.000846,"COR_P_F01":0.000002)[&"SH/boot"="81.7/100"]
:0.000853)[&"SH/boot"="0/85"]:0.000003)[&"SH/boot"="87.4/97"]:0.000885)[&"SH/boot"="0/86"]:0
.000002)[&"SH/boot"="62.7/94"]:0.000558)[&"SH/boot"="98.9/100"]:0.009159,("COR_K_F08":0.003
812,("COR_H_M02":0.004644,("COR_D_M05":0.00175,(("COR_K_F04":0.000002,(("COR_D_F03":0.0
00002,"COR_G_F03":0.002708)[&"SH/boot"="83.6/100"]:0.000853,(("COR_G_F01":0.002557,"COR_
G_F04":0.000002)[&"SH/boot"="86.3/98"]:0.000834,("COR_K_F03":0.001725,("COR_G_M04":0.0016
67,"COR_Z_F05":0.003576)[&"SH/boot"="0/86"]:0.000003)[&"SH/boot"="85.3/93"]:0.000868)[&"SH
/boot"="0/8"]:0.000003)[&"SH/boot"="0/5"]:0.000002)[&"SH/boot"="0/5"]:0.000002,(("COR_K_F01
":0.000852,("COR_Z_F02":0.001755,("COR_D_M04":0.000002,"COR_P_M03":0.000002)[&"SH/boot"
="91.6/100"]:0.000868)[&"SH/boot"="0/73"]:0.000003)[&"SH/boot"="84.1/98"]:0.000853,("COR_G_

M05":0.001861,(("COR_G_M01":0.002702,"COR_Z_M01":0.003511)[&"SH/boot"="0/36"]:0.000002,(
"COR_P_F02":0.001766,"COR_Z_M04":0.000897)[&"SH/boot"="0/31"]:0.000002)[&"SH/boot"="0/37
"]:0.000002)[&"SH/boot"="84/87"]:0.000874)[&"SH/boot"="0/13"]:0.000003)[&"SH/boot"="0/7"]:0.
000002)[&"SH/boot"="0/30"]:0.000003)[&"SH/boot"="77.4/88"]:0.001726)[&"SH/boot"="90.6/97"]:
0.003805)[&"SH/boot"="90.7/100"]:0.002721)[&"SH/boot"="89.1/91"]:0.006728)[&"SH/boot"="83.6
/97"]:0.006868)[&"SH/boot"="99.8/100"]:0.110255)[&"SH/boot"="97.7/97"]:0.035338)[&"SH/boot"
="93.8/70"]:0.140451)[&"SH/boot"="100/100"]:0.223725)[&"SH/boot"="78.3/58"]:0.078968)[&"SH/
boot"="96.3/99"]:0.080004)[&"SH/boot"="66/86"]:0.047239):0.0;

end;
